# Computation-Based Discovery of Potential Targets for Rheumatoid Arthritis and Related Molecular Screening and Mechanism Analysis of Traditional Chinese Medicine

**DOI:** 10.1155/2022/1905077

**Published:** 2022-06-04

**Authors:** Jijia Sun, Baocheng Liu, Ruirui Wang, Ying Yuan, Jianying Wang, Lei Zhang

**Affiliations:** ^1^Shanghai Collaborative Innovation Center of Traditional Chinese Medicine Health Service, Shanghai University of Traditional Chinese Medicine, Shanghai 201203, China; ^2^Department of Mathematics and Physics, School of Pharmacy, Shanghai University of Traditional Chinese Medicine, Shanghai 201203, China; ^3^Teaching and Research Section of Chinese Materia Medica, School of Pharmacy, Shanghai University of Traditional Chinese Medicine, Shanghai 201203, China

## Abstract

This study is aimed at screening potential therapeutic ingredients in traditional Chinese medicine (TCM) and identifying the key rheumatoid arthritis (RA) targets using computational simulations. Data for TCM-active ingredients with clear pharmacological effects were collected. Absorption, distribution, metabolism, excretion, and toxicity were evaluated. Potential RA targets were identified using the Gene Expression Omnibus (GEO) database, protein–protein interaction network, and Kyoto Encyclopedia of Genes and Genomes (KEGG) pathway enrichment analyses and potential TCM ingredients using AutoDock Vina. To examine the mechanisms underlying small molecules, target prediction, Gene Ontology, KEGG, and network modeling analyses were conducted; the effects were verified in rat synovial cells using cell proliferation assay. The activities of tumor necrosis factor TNF-*α* and IL-1*β* and alterations in cellular target protein levels were detected by ELISA and Western blotting, respectively. In total, data for 432 TCM active ingredients with clear pharmacological effects were obtained. Five critical RA-related genes were identified; CCL5 and CXCL10 were selected for molecular docking. Target prediction and network-based proximity analysis showed that dioscin could modulate 22 known RA clinical targets. Dioscin, asiaticoside, and ginsenoside Re could effectively inhibit *in vitro* cell proliferation and secretion of TNF-*α* and IL-1*β* in RA rat synovial cells. Using bioinformatics and computer-aided drug design, the potential small anti-RA molecules and their mechanisms of action were comprehensively identified. Dioscin could significantly inhibit proliferation and induce apoptosis in RA rat synovial cells by reducing TNF-*α* and IL-1*β* secretion and inhibiting abnormal CCL5, CXCL10, CXCR2, and IL2 expression.

## 1. Introduction

Rheumatoid arthritis (RA) is a chronic, systemic, inflammatory, and progressive autoimmune disease that affects synovial joints and other organ systems [1]. To date, the underlying disease mechanism of RA remains unclear, but it is generally believed to be initiated by infection and inflammatory mediators. Furthermore, recent studies have shown that the pathogenesis of RA is related to the changes and effects of genetic, bacterial, and viral factors; T and B lymphocytes; cytokines; and other immune cells [2, 3]. The incidence of RA increases with age. Approximately 0.3% to 1.0% of the population worldwide are affected by RA each year. Furthermore, RA is more common in adults aged 35–50 years, and the incidence in women is approximately three times that in men [4, 5].

Currently, the available treatment of RA includes nonsteroidal anti-inflammatory drugs, glucocorticoid drugs, and biological macromolecular therapy; however, they are costly and often exert toxicity and cause side effects [6, 7]. Therefore, further improvement of the efficacy and safety of anti-RA drugs and reduction of the costs is necessary.

Traditional Chinese medicine (TCM) has a long history of RA treatment based on rich experiences in clinical applications. RA, a common type of arthritis, belongs to “Bi syndrome” category in Chinese medicine [8]. Currently, Chinese medicines and compound prescriptions against RA have shown anti-inflammatory, analgesic, immunomodulatory, multilevel, and multistep therapeutic effects and present advantages of high safety, few adverse reactions, and cost effectiveness. Therefore, they have potential application for the treatment of RA [9].

**Figure 1:**
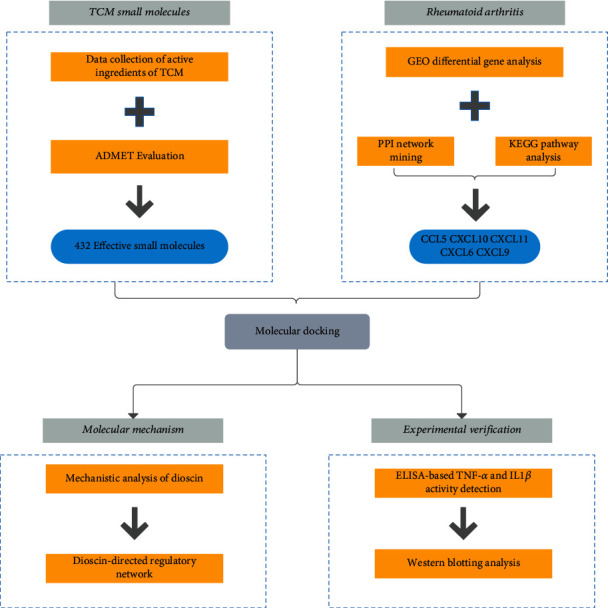
Workflow of computation-based discovery of potential targets for RA and related molecular screening and mechanism analysis of TCM.

Although several TCM compounds have been reported to exert good curative effects and minor side effects, the discovery of additional potential anti-RA ingredients from a large number of common small TCM molecules with clear pharmacological effects has important practical significance. In this view, we integrated bioinformatics, computer-aided drug design, and *in vitro* cellular experiments, in combination with existing literature analysis, to explore important targets for the treatment of RA and further screen out the active ingredients of TCM with potential therapeutic effects. *In vitro* experiments were performed to confirm these findings, with the aim of providing powerful methods and technical support for the treatment of RA with TCM. The workflow of this study is shown in Figure 1.

## 2. Materials and Methods

### 2.1. Data Collection

The active ingredients of common TCMs that have been proven to have clear pharmacological effects were collected from published literature in Chinese [10, 11]. The PubChem database (https://pubchem.ncbi.nlm.nih.gov/) was used to confirm the chemoinformatics data for each ingredient, including molecular name CAS, PubChem CID, molecular formula, canonical SMILES, and SDF files. A more authoritative and reliable database of active ingredients in commonly used Chinese medicines has been constructed.

The ACD/Labs software and SwissADME online system (http://www.swissadme.ch/) [12] were used to perform absorption, distribution, metabolism, excretion, and toxicity (ADMET) evaluation and analysis of all small molecules of TCM. Twelve ADMET evaluation indicators were screened, including Lipinski, molecular weight, log*P*, solubility, blood–brain-barrier (BBB) permeant, Pgp substrate, GI absorption, bioavailability score, synthetic accessibility, metabolic stability, Ames test, and hERG.

### 2.2. Gene Expression Omnibus (GEO) Differential Gene Analysis for RA

In the GEO database (https://www.ncbi.nlm.nih.gov/geo/), “rheumatoid arthritis” was queried to download the gene expression profile chip data related to RA. Perl language (version 5.32.0) was used to perform preprocessing of the original data, such as standardization, correction, and gene name annotation. The R language-based limma package [13] (version 3.44.0) was used for differentially expressed gene (DEG) analysis, and the upregulated and downregulated DEGs in each set of chip data were screened out when  |log2 FC| > 1.0 and adj.*p*.value < 0.05.

### 2.3. Construction of Disease-Associated Protein–Protein Interaction (PPI) Network and Discovery of Key Targets

All selected genes were imported into the STRING database (version 11.0, https://string-db.org/) to obtain the PPIs of the DEGs. The parameters were set as follows: organism, *Homo sapiens*; combined score threshold, 0.7. In addition, Gephi software (Version: 0.9.2, https://gephi.org/) was used to visualize the PPI network, and cytoHubba in Cytoscape (version 3.7.1, https://cytoscape.org/) [14], in which the algorithm selects the maximal clique centrality (MCC), was used to screen out the key targets in the PPI network.

### 2.4. Gene Ontology (GO) Function Annotation and Kyoto Encyclopedia of Genes and Genomes (KEGG) Pathway Enrichment Analysis

The GO database constructed by the GO Consortium in 2000 contains information for the biological process (BP), molecular function (MF), and cellular component (CC) of genes [15]. A biological process or pathway involves a group of genes functioning together. Enrichment analysis is used to identify classes of overrepresented genes or proteins, and a few of these may have an association with disease phenotypes. GO analysis uses statistical approaches to identify significantly enriched or depleted groups of genes.

In this study, clusterProfiler (version 3.14.3) [16] was used to perform GO function annotation and KEGG pathway enrichment analysis on all differential gene sets in RA, which were screened according to *p* value < 0.05 and *q* value < 0.05.

### 2.5. Screening of Small Molecules of TCM Based on Key Targets and Molecular Docking

The key protein targets in the PPI network were identified, and the targets in the reported RA-related pathways were obtained from the KEGG pathway enrichment analysis as described in Sections 2.3 and 2.4, respectively. The common targets from the two above-mentioned gene sets were considered the potential key targets of RA.

The virtual screening process of small molecules of anti-RA TCM was based on the molecular docking method, which involved the following steps:

1. The PDB file for the potential key target proteins was downloaded from the PDB database (http://www.rcsb.org/). PyMOL (version 1.7.0, https://pymol.org/) was used to evaluate key target proteins to remove water molecules, impurities due to the presence of ions, and perform other changes. AutoDockTools (version 1.5.6, http://autodock.scripps.edu/wiki/AutoDockTools) was used to hydrogenate and charge the processed protein-ligand; the processed PDB files were converted and saved in the PDBQT file format
2. The information of small molecules was downloaded from PubChem in two SDF format files, 2D and 3D. OpenBabel (version 2.4.0, http://openbabel.org/) was used to convert the 2D SDF file into a 3D structure file. The original 3D structure was directly converted into a PDB file, and all small molecules were processed with minimum energy; finally, the PDB file was converted into a PDBQT file
3. To determine the docking center parameters, we referred to the binding site (region) of the protein receptor and the original ligand. The box size was defined as 30 × 30 × 30, and AutoDock Vina (version 1.1.2, http://vina.scripps.edu/) was used to perform a semiflexible molecular docking and calculate the affinity values (kcal/mol) of all small molecules with potential key targets. Generally, the lower the affinity value, the greater the possibility that the small molecule binds to the receptor; therefore, according to the affinity value, the small molecules were sorted from small to large, and small molecules of TCM with potential anti-RA activity were screened out

### 2.6. In-Depth Research on the Mechanism of Small Molecules of TCM Obtained through Screening

To examine the underlying mechanisms of small molecules of anti-RA TCM more comprehensively, we further used the target prediction method to discover other potential targets and combined the findings with those of the abovementioned bioinformatics analysis and molecular docking screening.

Corresponding canonical SMILES of the obtained small molecules were imported into online prediction systems, such as HitPick (http://mips.helmholtz-muenchen.de/proj/hitpick) [17], SEA (http://sea.bkslab.org/) [18], SwissTargetPrediction (http://www.swisstargetprediction.ch/) [19], and STITCH (version 5.0, http://stitch.embl.de/), to predict their potential targets. In particular, the screening threshold precision for HitPick was set at 50%, the threshold MaxTc for SEA was set at 0.7, and the threshold probability for SwissTargetPrediction was set at 0.15. Molecular docking verification, GO function annotation, and KEGG pathway enrichment analyses were performed on the predicted potential targets.

### 2.7. Analysis of the Regulation of Known RA Treatment Targets by Small Molecules of TCM Based on Network Proximity

Next, we determined whether the small molecules of TCM screened have direct or indirect regulatory effects on the clinically known RA treatment targets. Given that some of these molecules may not directly act on certain RA targets but regulate the disease via the intervention of its neighboring targets, we further analyzed the small molecules of TCM using the calculation method of network proximity, based on the human PPI background network [20], using the following formula [21]:

(1) $$\begin{array}{c} \langle d_{AB}\rangle =\frac{1}{\left\|A\right\|\times \left\|B\right\|}\underset{a\in A,b\in B}{\sum }d\left(a,b\right),\\ S_{AB}=\langle d_{AB}\rangle -\frac{\langle d_{AA}\rangle +\langle d_{BB}\rangle }{2}, \end{array}$$

where *A* is the target set of small molecules of TCM, ‖*A*‖ is the number of the targets of *A*, *B* is the clinically known set of RA treatment targets, ‖*B*‖ is the number of the targets of *B*, *d*(*a*, *b*) is the shortest path distance between two nodes in the human PPI background network, 〈*d*_*AA*_〉 represents the average distance between the target points of the component action, 〈*d*_*BB*_〉 represents the average distance between the key genes, and 〈*d*_*AB*_〉 represents the average distance between the small Chinese medicine molecular target set and the clinically known RA treatment target set on the background network.

Generally, when *S*_*AB*_ < 0, the small molecule target *A* of TCM and the clinically known RA treatment target set *B* are close in network topology, indicating that the small molecule of TCM can interfere with the target set *A* and regulate the clinically known RA treatment target set *B*. When *S*_*AB*_ ≥ 0, *A* and *B* are separated in the network topology, indicating that the small molecule of TCM has no significant regulatory intervention on *B*. Therefore, we can calculate the value of *S*_*AB*_ to further judge whether the small molecules of TCM can interfere with the known clinical disease target set by regulating some other target proteins to further improve the treatment of the disease.

For identifying the clinically known RA treatment targets, we queried across two databases: Therapeutic Target Database (TTD, http://db.idrblab.net/ttd/) and DrugBank (https://www.drugbank.ca/). Thereafter, the intersection of the RA targets collected using the two databases was used to construct the final known RA clinical treatment target set.

In this study, we used R language (version 3.6.2) and igraph (version 1.2.5) to complete the aforementioned programming, calculations, and analyses.

### 2.8. Verification of the Targets through *In Vitro* Cell Experiments

#### 2.8.1. Materials, Reagents, and Instruments

*Materials:* rat synovial cells were induced using type II collagen and isolated in our laboratory.

*Reagents:* PBS and RPMI 1640 medium were purchased from Gibco (Grand Island, NY, U.S.A.); fetal bovine serum (FBS) was purchased from Biological Industries (Kibbutz Beit Haemek, Israel); antibiotic-antimycotic, bovine serum albumin, and trypsin-EDTA (0.05%) were purchased from Life Technologies (Carlsbad, California, U.S.A.); rat TNF-*α* and interleukin IL-1*β* enzyme-linked immunosorbent assay (ELISA) kits were purchased from LinkTech (Nanjing, China).

*Instruments:* EPOCH type multifunctional microplate reader (BioTek, Winooski, VT, USA), 3111 CO_2_ incubator (Thermo, Waltham, MA, USA), CX23 microscope (Olympus, Tokyo, Japan), and HFsafe-1200LC biological safety cabinet (Likang, Hong Kong, China) were used for the *in vitro* studies.

#### 2.8.2. Effect of Small Molecules on the Proliferation of Normal Membrane Cells and RA Synovial Cells

Synovial cells were cultured in RPMI-1640 medium supplemented with 10% FBS. After counting, the cells were seeded in 96-well culture plates at a density of 2000 cells/well. The cells were incubated with different concentrations of the small molecules of TCM. After 72 h, the culture medium was discarded; 90 *μ*l of fresh culture medium and 10 *μ*l of Cell Counting Kit-8 (CCK-8) reagent were added to the wells. After incubation at 37°C for 4 h, the absorbance at 450 nm was measured with a microplate reader to calculate the IC_50_ values using Graph Pad (Version: 8.0) software.

#### 2.8.3. Detection of TNF-*α* and IL-1*β*

The collagen II-induced arthritis (CIA) rat synovial cells were maintained and cultured in RPMI-1640 medium containing 10% FBS, 100 U/ml penicillin, and 100 U/ml streptomycin and were seeded in a 6-well culture plate at a density of 4 × 10^5^ cells/well. A total of 1800 *μ*l of RPMI-1640 serum-containing culture medium per well was added to the culture, with an additional 200 *μ*l drug solution. The final concentration of each sample was calculated as the IC_50_ concentration of the cells. The drug-free group was used as the arthritis model cell control group and cultured at 37°C in a 5% CO_2_ incubator for 72 h. The culture medium was collected, centrifuged at 400 × *g* for 5 min, and the supernatant was collected. ELISA was used for the detection of TNF-*α* and IL-1*β*, according to the manufacturer's instructions.

#### 2.8.4. Detection of the Effects of Small Molecules of TCM on Target Proteins through Western Blotting

The sample proteins collected as described in Section 2.8.3 were boiled at 100°C for 5 min. Thereafter, equal amounts of protein from each sample were loaded on a 10% SDS-PAGE gel for electrophoresis. After transferring the proteins on the gel to a cellulose acetate membrane with transfer buffer, the blots were blocked with 5% skim milk powder for 1 h. The blots were then incubated with different concentrations of primary antibody overnight at 4°C, followed by TBS-T washes and incubation with horseradish peroxidase-labeled secondary antibody. ECL was used to develop chemiluminescence, and the images were captured.

## 3. Results

### 3.1. Data Collection for Active Ingredients of TCM and ADMET Analysis

After collection of published literature data and confirmation from the PubChem database, chemoinformatic data of 432 effective small molecules of TCM and their corresponding 3D structure SDF files were obtained (Supporting material 1). For small molecules without 3D structure files, first, the 2D structure files were downloaded, and then, OpenBabel was used to convert them into a 3D structure file. The MMFF94 force field was applied for energy optimization.

**Figure 2:**
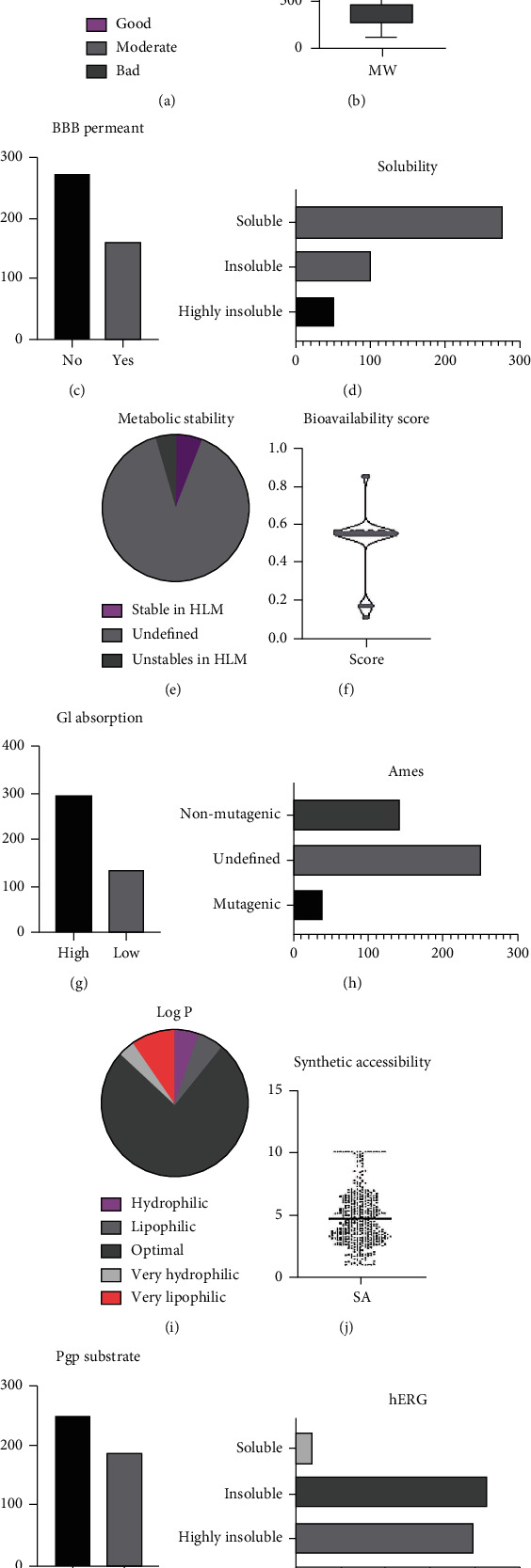
ADMET evaluation results of 432 effective small molecules of TCM based on ACD/Labs software and SwissADME. (a) Pie chart of Lipinski drug property evaluation; (b) Box plot showing statistical results for molecular weight; (c) Statistical results for BBB permeant; (d) Statistical results for solubility; (e) Pie chart showing statistical results for metabolic stability; (f) Violin graph of statistical results for bioavailability score; (g) Statistical results for GI absorption; (h) Statistical results for Ames, (i) Pie chart showing statistical results for lipophilicity (log*P*); (j) Violin chart showing statistical distribution of synthetic accessibility; (k) Statistical results for Pgp substrate; (l) Statistical results for hERG.

The canonical SMILES of 432 small molecules of TCMs were input into the ACD/Labs software and SwissADME for ADMET prediction evaluation. The results are shown in Figure 2. The evaluation results showed that among the 432 small molecules collected, 275 showed good Lipinski properties, 70 had moderate (moderate), and 87 exhibited low scores. The average, minimum, and maximum molecular weights of all small molecules were 395.05, 103.12, and 1468.52, respectively. In total, 160 small molecules were BBB permeant, 278 had good water solubility, and 164 were of poor solubility. A total of 24 small molecules showed high metabolic stability; however, a few showed temporary uncertainty, and 20 small molecules showed potential metabolic instability. Most small molecules had a good bioavailability score; only a few scored less than 0.55. Genotoxicity prediction (through Ames test) showed that 39 small molecules had potential mutagenicity, and 142 were clearly nontoxic; however, mutagenicity of 297 molecules could not be determined. The intestinal absorption of each small molecule was high. The lipophilicity (log*P*) evaluation showed that 330 small molecules exhibited optimal lipophilicity (optimal). The average value for the synthetic accessibility of small molecules was 4.69, suggesting that most compounds were easy to synthesize in the laboratory. The prediction of cardiac inhibitory toxicity (hERG) suggested that 19 small molecules had cardiotoxic properties (Supporting material 2).

### 3.2. RA Chip Data Collection and Differential Gene Analysis

GSE55235, GSE55457, and GSE77298 were obtained from the GEO query to obtain the expression profile data related to RA. The platform of GSE55235 is GPL96 [HG-U133A] Affymetrix Human Genome U133A Array, which contains 10 normal samples and 10 RA samples; GSE55457 uses GPL96 [HG-U133A] Affymetrix Human Genome U133A Array as the platform, which contains 10 normal samples and 13 RA samples; the platform of GSE77298 is GPL570 [HG-U133_Plus_2] Affymetrix Human Genome U133 Plus 2.0 Array, which contains seven healthy samples and 16 RA samples.

**Figure 3:**
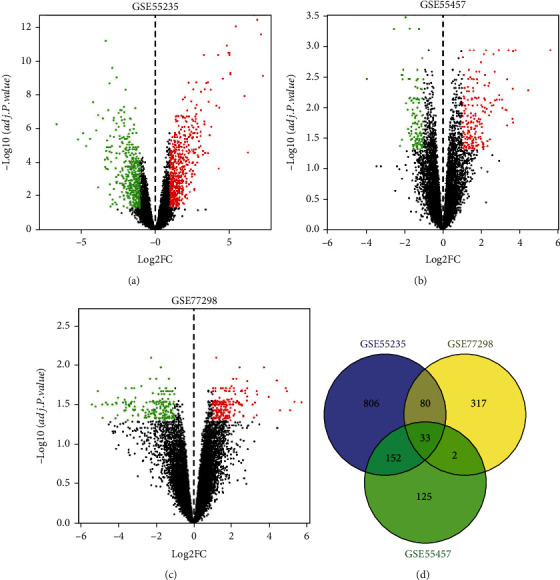
(a) Volcano map of GSE55235 DEG analysis; (b) volcano map of GSE55457 DEG analysis; (c) volcano map of GSE77298 DEG analysis; (d) Venn diagram of RA gene-based GEO differential gene analysis.

After differential gene analysis, 1071 DEGs were screened in GSE55235—599 were upregulated and 472 were downregulated; 312 DEGs were screened in GSE55457—189 were upregulated and 123 were downregulated; and 432 DEGs were screened in GSE77298—237 were upregulated and 195 were downregulated. The volcano diagram of GEO differential gene analysis of each group is shown in Figures 3(a)–3(c). In this study, we included the DEGs that concurrently appeared in any two chip expression datasets into the RA gene set. Therefore, a total of 267 RA-related DEGs were obtained, as shown in Figure 3(d).

### 3.3. PPI Network Construction and Screening of Key Targets

**Figure 4:**
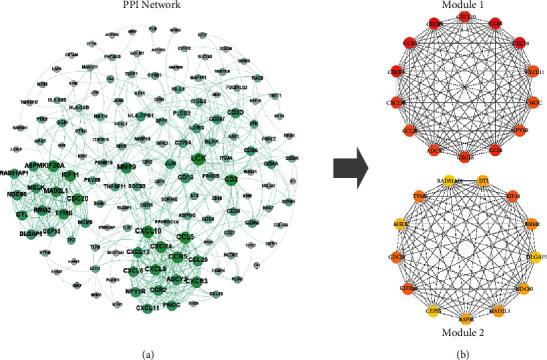
(a) PPI network of RA. The dots represent individual proteins. The greater the degree of the node, the darker the color, and the larger the size of the node. (b) The top 27 important proteins in the PPI networks are presented based on the MCC algorithm and 2 submodules they formed.

All 267 differential genes of RA were imported into the STRING database to obtain the corresponding PPI network according to the method described in Section 2.3, as shown in Figure 3(a). Thereafter, cytoHubba was utilized to screen out the top 10% of the important proteins (27 proteins) in the PPI network, namely, CXC chemokine receptor type 2 (CXCR4; degree = 17), CXCL9 (degree = 16), CCR5 (degree = 18), CXCR3 (degree = 16), CCR2 (degree = 14), CXCL10 (degree = 19), CCL5 (degree = 18), CXCL6 (degree = 14), CXCL13 (degree = 14), ADCY2 (degree = 14), CCL25 (degree = 14), PNOC (degree = 13), NPY1R (degree = 13), CXCL11 (degree = 13), KIF11 (degree = 17), KIF20A (degree = 17), CDC20 (degree = 17), TYMS (degree = 15), RRM2 (degree = 14), MAD2L1 (degree = 15), DTL (degree = 13), ASPM (degree = 13), NDC80 (degree = 13), MELK (degree = 13), RAD51AP1 (degree = 12), CEP55 (degree = 12), and DLGAP5 (degree = 12). These 27 significant proteins were divided into 2 submodules (module 1 and module 2) with high internal connections, as shown in Figures 4(a) and 4(b).

### 3.4. GO Function Annotation and KEGG Pathway Enrichment Analysis Results

**Figure 5:**
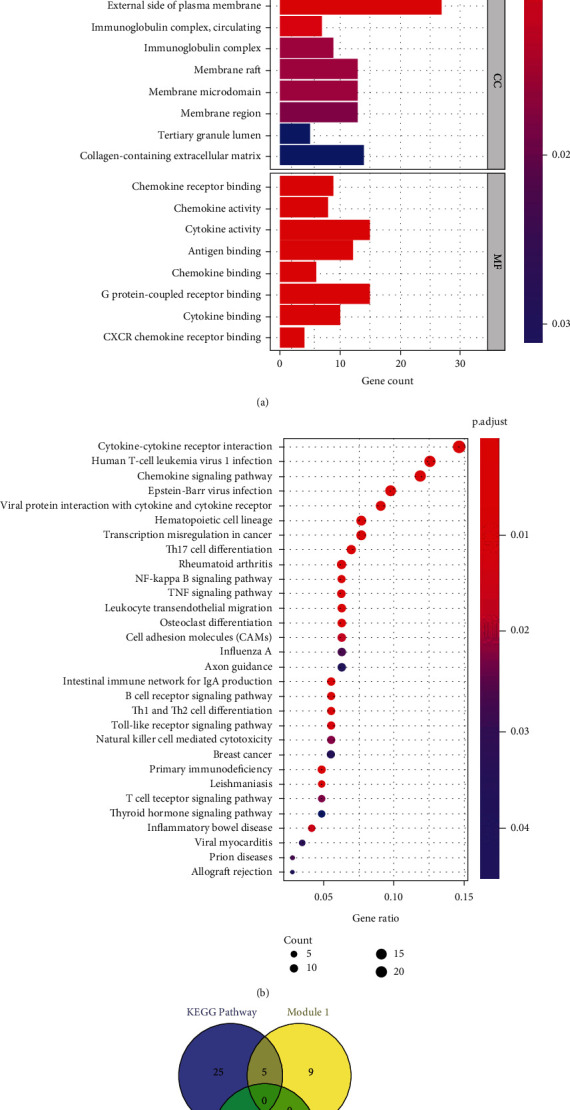
(a) GO function annotation of RA DEGs; (b) KEGG pathway enrichment analysis of RA DEGs; (c) the intersection of the targets in the main RA enrichment pathway and the two submodules of the important targets.

ClusterProfiler was used to perform GO function and KEGG pathway enrichment analyses on 267 differential gene sets of RA, where *p* value < 0.05 and *q* value < 0.05. GO function annotation, KEGG pathway enrichment analysis, and target-pathway enrichment network are presented in Figures 5(a) and 5(b).

GO function annotation indicated that these DEGs were mainly involved in 443 BP functions, such as regulation of lymphocyte activation (GO:0051249), leukocyte migration (GO:0050900), immune response activation of the cell surface receptor signaling pathway (GO:0002429), and antigen receptor-mediated signaling (GO:0050851). Thirteen CC functions mainly include the outer side of the plasma membrane (GO:0009897), extracellular matrix containing collagen (GO:0062023), and membrane rafts (GO:0045121). In addition, 19 MFs involved receptor ligand activity (GO:0048018), cytokine activity (GO:0005125), G-protein-coupled receptor binding (GO:0001664), and endopeptidase activity (GO:0004175), among several others.

KEGG pathway enrichment analysis revealed that RA-related DEGs mainly involved 31 related pathways. Among them, Th17 cell differentiation (hsa04659), rheumatoid arthritis (hsa05323), NF-kappa B signaling pathway (hsa04064), TNF signaling pathway (hsa04668), Th1 and Th2 cell differentiation (hsa04658), and Toll-like receptor signaling pathway (hsa04620) have been reported to be closely related to RA. A total of 30 targets enriched in these pathways were screened out, namely, BCL2A1, BIRC3, BLNK, CCL5, CD247, CD3D, CXCL10, CXCL11, CXCL6, CXCL9, GADD45B, HLA-DMB, HLA-DOB, HLA-DPB1, IL15, IL1R1, IL21R, IL2RG, ITGB2, JUN, JUNB, LCK, MMP9, PLCG2, PRKCB, SOCS3, SPP1, TLR7, TLR8, and TNFSF11, as shown in Table 1.

We further examined the intersection shown in Figure 5(c) and found that there were five targets associated with both the key proteins in the PPI network and the genes in the RA-related pathways. More interestingly, the target proteins CCL5, CXCL10, CXCL11, CXCL6, and CXCL9 appeared unbiasedly in module 1 of the PPI network. However, three of them do not exist in the PDB database without an appropriate PDB structure file; thus, we only used two proteins, CCL5 (CC motif chemokine 5, PDB ID: 5L2U) and CXCL10 (CXC motif chemokine 10, PDB ID: 1O7Y), with complete PDB information files for follow-up research.

### 3.5. Molecular Docking Analysis

**Figure 6:**
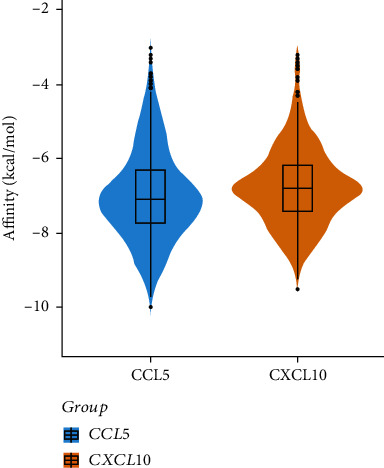
Violin diagram of molecular docking results of 432 small molecules of TCM with CCL5 and CXCL10.

AutoDock Vina was used to perform bulk molecular docking on the two protein targets CCL5 and CXCL10, and the affinity values of 432 small molecules of TCM to act on the targets were obtained, except for the very few unsuccessful dockings. The distribution of the molecular docking scores of all small molecules of TCM on CCL5 and CXCL10 is shown in Figure 6.

In this study, we only listed the top 10 small molecules of TCM with 15 molecular docking scores, as shown in Table 2. Table 2 demonstrates that ginsenoside Re, asiaticoside, ergotamine, neferine, polyphyllin II, dioscin, raddeanin A, berbamine, ginsenoside Rg1, and tubeimoside I, in turn, had better scoring results when docking with CCL5 than with CXCL10, while alpha-crocin, polyphyllin II, dioscin, digoxin, ergotamine, saikosaponin A, raddeanin A, polyphyllin VI, asiaticoside, and jujuboside A had higher scores for CXCL10 affinity than for CCL5.

**Figure 7:**
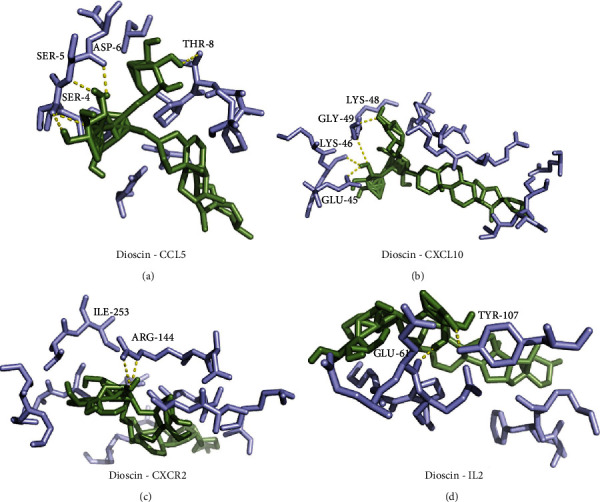
(a) Conformation of dioscin and CCL5; (b) dioscin and CXCL10; (c) dioscin and CXCR2; and (d) dioscin and IL2 using molecular docking. The yellow dashed lines indicate hydrogen bond interactions.

Among the small molecules, two saponins, polyphyllin II and dioscin, had affinities of less than -9.0. Based on molecular docking conformation analysis of these two small molecules, we hypothesized that a combination of dioscin with CCL5 and CXCL10 was most stable. Therefore, we selected dioscin for subsequent in-depth analysis, and the binding conformations of CCL5 and CXCL10 are shown in Figures 7(a) and 7(b).

### 3.6. Potential Target Prediction and Molecular Docking of Dioscin

To comprehensively analyze the potential anti-RA mechanism of dioscin, we used HitPick, SEA, SwissTargetPrediction, and STITCH to predict and screen the targets of dioscin. As a result, we found that dioscin had two potential targets, CXCR2 and IL2. In addition to the results of molecular docking screening presented in Section 3.5, four potential targets, CCL5, CXCL10, CXCR2, and IL2, were proposed for dioscin interaction.

Next, we verified the molecular docking of dioscin for CXCR2 (PDB ID: 6LFO) and IL2 (PDB ID: 4NEM). The results showed that the docking score of dioscin and CXCR2 was -9.1, which was identical to the docking score of dioscin and IL2 (-9.1). The molecular binding conformation also indicated that dioscin could form stable interactions with these two protein targets, as shown in Figures 7(c) and 7(d).

### 3.7. Mechanistic Analysis of the Anti-RA Effects of Dioscin

**Figure 8:**
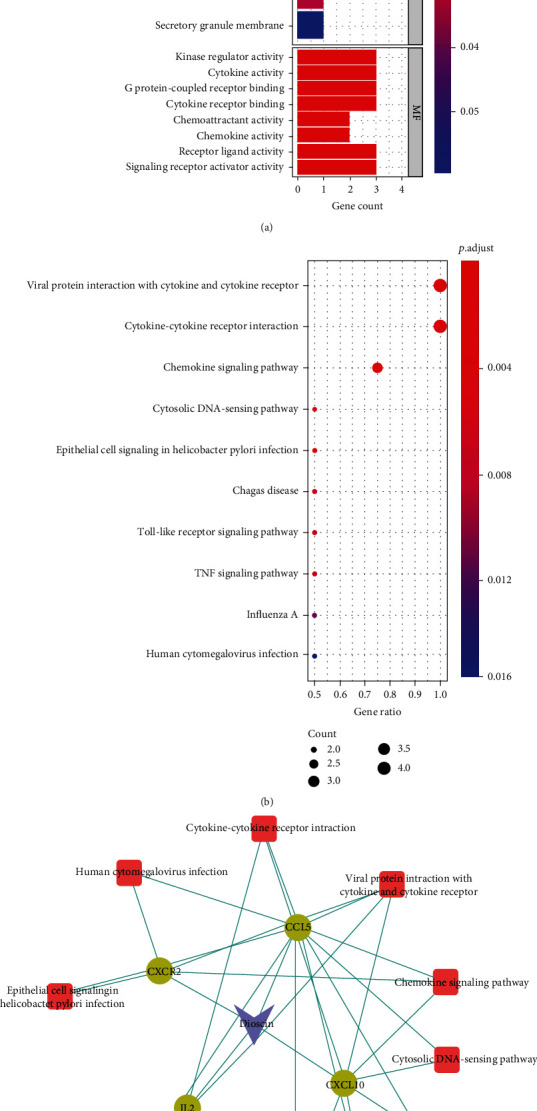
(a) GO function annotation results of the four targets of dioscin; (b) KEGG pathway enrichment analysis of the four targets of dioscin; (c) dioscin target-pathway enrichment network. Dots represent protein targets, squares represent pathways, and V-shape represents small molecules of TCM.

Furthermore, GO function annotation and KEGG pathway enrichment analysis revealed that the four targets CXCR2, CCL5, CXCL10, and IL2 were mainly involved in GO functions, such as cellular calcium homeostasis (GO:0006874), calcium homeostasis (GO:0055074), cellular divalent inorganic cation homeostasis (GO:0072503), kinase regulator activity (GO:0019207), cytokine activity (GO:0005125), G-protein-coupled receptor binding (GO:0001664), and external side of the plasma membrane (GO:0009897). The KEGG pathway was mainly enriched in 10 pathways. The interaction pathways between viral proteins and cytokines and cytokine receptors (hsa04061), the interaction pathway between cytokines and cytokine receptors (hsa04060), chemokine signaling pathway (hsa04062), cytoplasmic DNA sensing pathway (hsa04623), epithelial cell signaling pathway in Helicobacter pylori infection (hsa05120), Chagas disease pathway (hsa05142), Toll-like receptor signaling pathway (hsa04620), TNF signaling pathway (hsa04668), influenza A (hsa05164), and human cytomegalovirus infection pathway (hsa05163) are shown in Figure 8.

### 3.8. Analysis of the Network Regulation of Dioscin on Clinically Known Therapeutic Targets of RA

A total of 140 and 193 known RA targets were collected from the TTD and DrugBank databases, respectively. After the two sets were intersected, 23 common RA treatment targets were obtained: ABCG2, AKR1B1, ALOX5, CASP1, DHFR, DHODH, HRH4, IL2, JAK1, JAK2, JAK3, MMP9, NR3C1, PDE4A, PDE4B, PDE4D, PLA2G1B, PPARA, PTGS1, PTGS2, TLR7, TLR9, and TNF. Among them, IL2 is also a potential direct target of dioscin.

**Figure 9:**
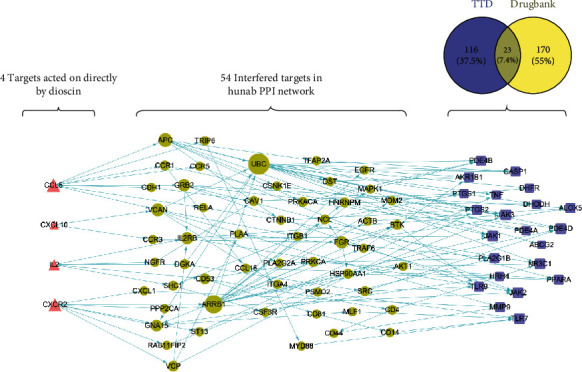
Dioscin-directed regulatory network. Dioscin can directly act on 4 targets (CCL5, CXCL10, CXCR2, and IL2), regulate 54 proteins in the human PPI network, and further target 22 RA treatment targets to exert therapeutic effects. The triangles represent the 4 targets directly affected by dioscin, the dots represent the 54 proteins in the human PPI network, and the squares represent the 22 known RA treatment targets.

Using the algorithm, we obtained the proximity values of dioscin to the 23 clinically known RA treatment targets on the human PPI network (*S*_*AB*_ = −1.16 < 0), where 〈*d*_*AB*_〉 = 2.85, 〈*d*_*AA*_〉 = 4.62, 〈*d*_*BB*_〉 = 3.40. Therefore, we speculated that dioscin could indirectly regulate and intervene in other 22 known RA targets by directly acting on the four targets, i.e., CCL5, CXCL10, CXCR2, and IL2, in treating RA. Based on further analysis of the human PPI network, we found that CCL5, CXCL10, CXCR2, and IL2 interacted with 54 proteins in the human PPI network to regulate other therapeutic targets of RA, as shown in Figure 9.

Furthermore, we searched “rheumatoid arthritis” on the TCMSP website (https://tcmspw.com/) and obtained 42 small molecules of TCM with anti-RA effects and corresponding therapeutic targets (Supporting material 3). Accordingly, the proximity *S*_*AB*_ of these 42 small Chinese medicine molecules on the human PPI network to 23 RA treatment targets was calculated. The results showed that the *S*_*AB*_ values of these 42 small molecules were all less than 0, which confirmed that dioscin had potential anti-RA effects.

### 3.9. Verification with *In Vitro* Cell Culture Experiments

Based on the results of molecular docking, we selected seven small molecules, namely, dioscin (250 *μ*g/ml), asiaticoside (300 *μ*g/ml), neferine (400 *μ*g/ml), ginsenoside Re (300 *μ*g/ml), polyphyllin II (800 *μ*g/ml), *Bupleurum* saikosaponin A (750 *μ*g/ml), and alpha-crocin I (1000 *μ*g/ml), for *in vitro* experiments. Tripterygium glycosides (150 *μ*g/ml) were used as a control for small molecules of TCM. Therefore, a total of eight small molecules of TCM were included in this experiment.

Cell proliferation experiments indicated that these eight small molecules of TCM had no significant effect on normal rat synovial cells, while the response of CIA rat synovial cells was significantly enhanced by dioscin, asiaticoside, and ginsenoside Re, with IC_50_ values of 230.1 ± 2.1 *μ*g/ml, 299.9 ± 2.8 *μ*g/ml, and 270.2 ± 2.5 *μ*g/ml, respectively.

**Figure 10:**
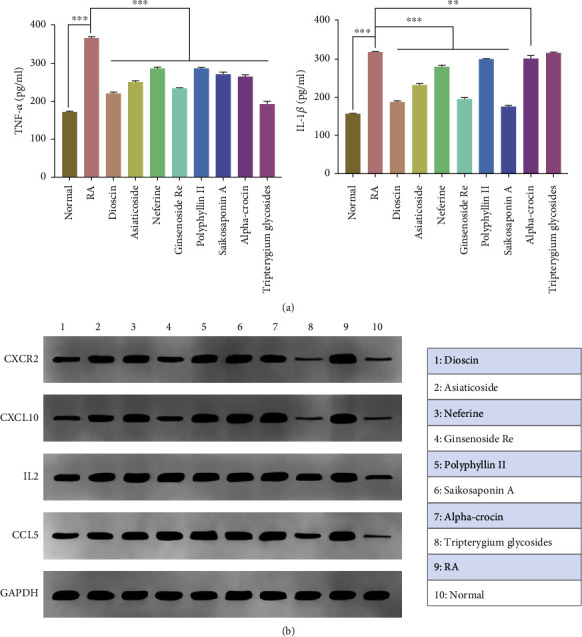
(a) Effects of different compounds on the expression of TNF-*α* and IL-1*β* in synovial cells of CIA rats (compared with RA group: ∗*p* < 0.05, ∗∗*p* < 0.01, ∗∗∗*p* < 0.001). (b) Relative expression results of CXCR2, CXCL10, IL2, and CCL5 in different groups detected through Western blotting.

Compared with the control group, the CIA rat synovial cells produced significantly increased levels of TNF-*α* and IL-1*β*. However, the TNF-*α* and IL-1*β* levels in the culture supernatant of the dioscin, asiaticoside, and ginsenoside Re groups were significantly reduced, which suggested that these TCMs could significantly inhibit the hypersecretion of TNF-*α* and IL-1*β* in CIA rat synovial cells (Figure 10(a)).

We further examined protein expression using Western blotting. Compared with the control and RA groups, the dioscin and asiaticoside groups showed significantly reduced expression of CCL5, while dioscin and ginsenoside Re groups showed significantly reduced expression of CXCL10. Similarly, CXCR2 expression was significantly inhibited in the dioscin, asiaticoside, and ginsenoside Re groups. Dioscin also had a certain inhibitory effect on IL2 expression. All the above-mentioned small molecules of TCM had similar effects to tripterygium glycosides, as shown in Figure 10(b).

## 4. Discussion

In this study, we first collected 432 effective common small molecules of TCM and screened out 267 RA-relevant genes from 3 sets of RA gene expression profile data. The PPI network based on differential genes enclosed important nodes. Using biological function enrichment analysis, we identified five critical genes related to RA. Combined with literature analysis and existing protein databases, two chemokines (CCL5 and CXCL10) were selected for follow-up research. Target prediction and *in vitro* experiment results collectively suggested that dioscin had a direct regulatory effect on CXCR2, CCL5, CXCL10, and IL2.

Reportedly, chemokines play an important role in inflammatory cell infiltration in the joints of patients with RA [22], and chemokine blockers are considered potential drugs for the treatment of RA [23]. Previous bioinformatics analyses have shown that the expression of CCL5 is closely related to RA [24]. CCL5 is produced by circulating T cells and plays an active role in the chemotactic activity of T cells in RA [25]. The expression of CXCL10 is increased in the RA serum and synovium. It not only plays an important role in the homing of chemotactic leukocytes in RA inflammation but can also destroy the bone tissue by activating nuclear factor-*κ*B ligands [26]. Studies have also found that the expression of CXCL10 in the peripheral blood and synovial fluid of RA patients is increased [27], and an increasing number of studies are now using CXCL10 as a novel target for RA treatment [28]. Similarly, the chemokine CXCR2 is known to be related to RA. CXCL1 promotes the expression of IL6 in synovial fibroblasts of osteoarthritis and RA patients via the CXCR2, c-Raf, MAPK, and AP-1 pathways [29]. Therapeutic blockade of CXCR2 can rapidly clear inflammation in arthritis and atopic dermatitis models [30]. Studies have shown that IL2 is a pleiotropic cytokine that can promote inflammation and maintain immune tolerance. Consequently, it is now an emerging therapeutic target for many anti-RA drugs [31].

Dioscin is found in medicinal plants such as *Dioscoreanigra*, *D. zingiberensis*, and *D. fuzhouensis*. Dioscin exerts many therapeutic effects on various diseases [32, 33], such as regulating the inhibitory effect of miR-125a-5p on STAT3 signaling and reducing glucose and lipid metabolism disorders in Type 2 diabetes (T2DM) suggesting that it exhibits anti-T2DM activity [34]. In an animal model of hyperuricemia, the effects of dioscin on the reduction of serum uric acid levels and the enhancement of uric acid excretion have been reported [35]. Moreover, dioscin inhibits the M2 polarization of macrophages in the JNK and STAT3 pathways, thereby triggering antitumor immunity [36]. Other studies have further shown that dioscin has similar pharmacokinetic characteristics to dexamethasone [37] and exerts antiarthritis effects by inhibiting the immune response of Th17 cells [38]. In addition, dioscin inhibits osteoclast differentiation and bone resorption by downregulating the Akt signaling cascade [39]. In our study, dioscin-targeted enrichment analysis of the KEGG pathway also showed that the resultant Toll-like receptor signaling pathway (hsa04620) and TNF signaling pathway (hsa04668) were the main signaling pathways related to RA. Therefore, accumulating evidence supports that, as a natural small molecule, dioscin has multiple therapeutic effects on RA and is worthy of in-depth study.

In this study, we used an algorithm based on network proximity to innovatively detect the regulation and intervention ability of dioscin on the known clinically therapeutic targets of RA. The results clearly showed that dioscin, by acting directly on four targets, indirectly intervenes and affects 54 related proteins and eventually regulates the main therapeutic targets of RA. We found that, in the network of dioscin-regulated RA targets, UBC (degree = 18), ARRB1 (degree = 14), APC (degree = 8), VCAN (degree = 7), GRB2 (degree = 6), IL2RB (degree = 6), FGR (degree = 6), and GNA15 (degree = 5) had higher degree values. The discovery of these target proteins in the network provides new insights for further related research and could serve as an important reference for the development of other RA drugs.

## 5. Conclusion

In summary, this study comprehensively used bioinformatics, molecular docking-based virtual screening, network modeling, and other computational methods; collected 432 commonly used TCM active ingredients; and analyzed the overall ADMET characteristics of these small molecules. The GEO and PPI network analyses helped to identify several key target genes associated with RA, and molecular docking technology was used to screen the small molecules of TCM for the treatment of RA. Furthermore, we analyzed the potential treatment mechanism of RA through network proximity-based prediction, and *in vitro* experiments confirmed that dioscin exerted significant regulatory effects on CCL5, CXCL10, CXCR2, and IL2. The series of integrated analysis methods performed in this study are expected to provide powerful technical support for the treatment of RA and development of novel molecules of TCM.

## Figures and Tables

**Table 1:** The main pathways and their corresponding enrichment targets that have been reported to be related to RA disease.

ID	Pathway	Genes
hsa04659	Th17 cell differentiation [40, 41]	*HLA-DMB, HLA-DOB, CD247, CD3D, LCK, IL1R1, JUN, IL21R, IL2RG, HLA-DPB1*
hsa05323	Rheumatoid arthritis	*HLA-DMB, HLA-DOB, CCL5, IL15, JUN, HLA-DPB1, ITGB2, CXCL6, TNFSF11*
hsa04064	NF-kappa B signaling pathway [42]	*GADD45B, BLNK, LCK, PRKCB, IL1R1, PLCG2, BIRC3, TNFSF11, BCL2A1*
hsa04668	TNF signaling pathway [43]	*CCL5, JUNB, SOCS3, IL15, JUN, BIRC3, CXCL10, CXCL6, MMP9*
hsa04658	Th1 and Th2 cell differentiation [44]	*HLA-DMB, HLA-DOB, CD247, CD3D, LCK, JUN, IL2RG, HLA-DPB1*
hsa04620	Toll-like receptor signaling pathway [45]	*CCL5, TLR7, JUN, CXCL9, CXCL11, TLR8, CXCL10, SPP1*

**Table 2:** Molecular docking results of the 432 small molecules with CCL5 and CXCL10 targets (top 10 ranking).

Receptor	Ligand	Molecular formula	PubChem CID	Affinity (kcal/Mol)
CCL5	Ginsenoside Re	C48H82O18	441921	-10.0
	Asiaticoside	C48H78O19	24721205	-9.7
	Ergotamine	C33H35N5O5	8223	-9.6
	Neferine	C38H44N2O6	159654	-9.6
	Polyphyllin II	C44H70O16	46200821	-9.5
	Dioscin	C45H72O16	119245	-9.4
	Raddeanin A	C47H76O16	174742	-9.3
	Berbamine	C37H40N2O6	275182	-9.2
	Ginsenoside Rg1	C42H72O14	441923	-9.1
	Tubeimoside I	C63H98O29	51346132	-9.1
CXCL10	Alpha-Crocin	C44H64O24	5281233	-9.5
	Polyphyllin II	C44H70O16	46200821	-9.2
	Dioscin	C45H72O16	119245	-9.0
	Digoxin	C41H64O14	2724385	-9.0
	Ergotamine	C33H35N5O5	8223	-8.9
	Saikosaponin A	C42H68O13	167928	-8.8
	Raddeanin A	C47H76O16	174742	-8.8
	Polyphyllin VI	C39H62O13	10417550	-8.7
	Asiaticoside	C48H78O19	24721205	-8.6
	Jujuboside A	C58H94O26	51346169	-8.6

## Data Availability

The datasets used and/or analyzed during the current study are available from the corresponding author on reasonable request.

## Supplementary Materials

Supplementary 1 Supporting material 1: information on 432 small molecules of TCM.

Supplementary 2 Supporting material 2: ADMET evaluation results of 432 small molecules of TCM.

Supplementary 3 Supporting material 3: the SAB values of these 42 small molecules of TCM.

## References

[1] Sparks J. A. (2019). Rheumatoid arthritis. *Annals of Internal Medicine*.

[2] Smolen J. S., Aletaha D., McInnes I. B. (2016). Rheumatoid arthritis. *Lancet*.

[3] Smolen J. S., Aletaha D., Barton A. (2018). Rheumatoid arthritis. *Nature Reviews. Disease Primers*.

[4] Charles J., Britt H., Pan Y. (2013). Rheumatoid arthritis. *Australian Family Physician*.

[5] Favalli E. G., Biggioggero M., Crotti C., Becciolini A., Raimondo M. G., Meroni P. L. (2019). Sex and management of rheumatoid arthritis. *Clinical Reviews in Allergy and Immunology*.

[6] Feng Z., Xu J., He G. (2016). The efficacy and safety of the combination of total glucosides of peony and leflunomide for the treatment of rheumatoid arthritis: a systemic review and meta-analysis. *Evidence-based Complementary and Alternative Medicine*.

[7] Strangfeld A., Richter A., Siegmund B. (2017). Risk for lower intestinal perforations in patients with rheumatoid arthritis treated with tocilizumab in comparison to treatment with other biologic or conventional synthetic DMARDs. *Annals of the Rheumatic Diseases*.

[8] Wang W., Wang X., Tang X., Jiang Q., Fan Y. (2019). Classifying rheumatoid arthritis by traditional Chinese medicine Zheng: a multi-center cross-sectional study. *Journal of Traditional Chinese Medicine*.

[9] Zhang P., Li J., Han Y., Yu X. W., Qin L. (2010). Traditional Chinese medicine in the treatment of rheumatoid arthritis: a general review. *Rheumatology International*.

[10] Qiang L. (2008). *Newly Compiled Manual of Effective Ingredients of Commonly Used Chinese Medicines*.

[11] Yubin J. (2011). *Pharmacology and Application of Active Ingredients in Traditional Chinese Medicine/People’s Medical Publishing House*.

[12] Daina A., Michielin O., Zoete V. (2017). SwissADME: a free web tool to evaluate pharmacokinetics, drug-likeness and medicinal chemistry friendliness of small molecules. *Scientific Reports*.

[13] Diboun I., Wernisch L., Orengo C. A., Koltzenburg M. (2006). Microarray analysis after RNA amplification can detect pronounced differences in gene expression using limma. *BMC Genomics*.

[14] Chin C. H., Chen S. H., Wu H. H., Ho C. W., Ko M. T., Lin C. Y. (2014). cytoHubba: identifying hub objects and sub-networks from complex interactome. *BMC Systems Biology*.

[15] Ashburner M., Ball C. A., Blake J. A. (2000). Gene Ontology: tool for the unification of biology. *Nature Genetics*.

[16] Yu G., Wang L. G., Han Y., He Q. Y. (2012). clusterProfiler: an R package for comparing biological themes among gene clusters. *OMICS*.

[17] Liu X., Vogt I., Haque T., Campillos M. (2013). HitPick: a web server for hit identification and target prediction of chemical screenings. *Bioinformatics*.

[18] Keiser M. J., Roth B. L., Armbruster B. N., Ernsberger P., Irwin J. J., Shoichet B. K. (2007). Relating protein pharmacology by ligand chemistry. *Nature Biotechnology*.

[19] Gfeller D., Grosdidier A., Wirth M., Daina A., Michielin O., Zoete V. (2014). SwissTargetPrediction: a web server for target prediction of bioactive small molecules. *Nucleic Acids Research*.

[20] Cheng F., Kovács I. A., Barabási A. L. (2019). Network-based prediction of drug combinations. *Nature Communications*.

[21] Menche J., Sharma A., Kitsak M. (2015). Disease networks. Uncovering disease-disease relationships through the incomplete interactome. *Science*.

[22] McInnes I. B., Schett G. (2011). The pathogenesis of rheumatoid arthritis. *The New England Journal of Medicine*.

[23] Nanki T. (2016). Treatment for rheumatoid arthritis by chemokine blockade. *Nihon RinshoMeneki Gakkai Kaishi.*.

[24] Li W. C., Bai L., Xu Y. (2019). Identification of differentially expressed genes in synovial tissue of rheumatoid arthritis and osteoarthritis in patients. *Journal of Cellular Biochemistry*.

[25] Luterek-Puszyńska K., Malinowski D., Paradowska-Gorycka A., Safranow K., Pawlik A. (2017). CD28, CTLA-4 and CCL5 gene polymorphisms in patients with rheumatoid arthritis. *Clinical Rheumatology*.

[26] Lee E. Y., Lee Z. H., Song Y. W. (2009). CXCL10 and autoimmune diseases. *Autoimmunity Reviews*.

[27] Proost P., Struyf S., Loos T. (2006). Coexpression and interaction of CXCL10 and CD26 in mesenchymal cells by synergising inflammatory cytokines: CXCL8 and CXCL10 are discriminative markers for autoimmune arthropathies. *Arthritis Research & Therapy*.

[28] Salomon I., Netzer N., Wildbaum G., Schif-Zuck S., Maor G., Karin N. (2002). Targeting the function of IFN-gamma-inducible protein 10 suppresses ongoing adjuvant arthritis. *Journal of Immunology*.

[29] Hou S. M., Chen P. C., Lin C. M., Fang M. L., Chi M. C., Liu J. F. (2020). CXCL1 contributes to IL-6 expression in osteoarthritis and rheumatoid arthritis synovial fibroblasts by CXCR2, c-Raf, MAPK, and AP-1 pathway. *Arthritis Research & Therapy*.

[30] Alam M. J., Xie L., Ang C. (2020). Therapeutic blockade of CXCR2 rapidly clears inflammation in arthritis and atopic dermatitis models: demonstration with surrogate and humanized antibodies. *MAbs*.

[31] Wu R., Li N., Zhao X. (2020). Low-dose interleukin-2: biology and therapeutic prospects in rheumatoid arthritis. *Autoimmunity Reviews*.

[32] Tao X., Yin L., Xu L., Peng J. (2018). Dioscin: a diverse acting natural compound with therapeutic potential in metabolic diseases, cancer, inflammation and infections. *Pharmacological Research*.

[33] Yang L., Ren S., Xu F., Ma Z., Liu X., Wang L. (2019). Recent advances in the pharmacological activities of dioscin. *BioMed Research International*.

[34] Xu L. N., Yin L. H., Jin Y. (2020). Effect and possible mechanisms of dioscin on ameliorating metabolic glycolipid metabolic disorder in type-2-diabetes. *Phytomedicine*.

[35] Zhang Y., Jin L., Liu J. (2018). Effect and mechanism of dioscin from _Dioscorea spongiosa_ on uric acid excretion in animal model of hyperuricemia. *Journal of Ethnopharmacology*.

[36] Cui L., Yang G., Ye J. (2020). Dioscin elicits anti-tumour immunity by inhibiting macrophage M2 polarization via JNK and STAT3 pathways in lung cancer. *Journal of Cellular and Molecular Medicine*.

[37] Marahatha R., Gyawali K., Sharma K. (2021). Pharmacologic activities of phytosteroids in inflammatory diseases: mechanism of action and therapeutic potentials. *Phytotherapy Research*.

[38] Cao Y. J., Xu Y., Liu B. (2019). A steroidal saponin isolated from Dioscorea nipponica, attenuates collagen-induced arthritis by inhibiting Th17 cell response. *The American Journal of Chinese Medicine*.

[39] Qu X., Zhai Z., Liu X. (2014). Dioscin inhibits osteoclast differentiation and bone resorption though down- regulating the Akt signaling cascades. *Biophys Res Commun*.

[40] Roeleveld D. M., van Nieuwenhuijze A. E., van den Berg W. B., Koenders M. I. (2013). The Th17 pathway as a therapeutic target in rheumatoid arthritis and other autoimmune and inflammatory disorders. *BioDrugs*.

[41] Bedoya S. K., Lam B., Lau K., Larkin J. (2013). Th17 cells in immunity and autoimmunity. *Clinical & Developmental Immunology*.

[42] Jimi E., Fei H., Nakatomi C. (2019). NF-*κ*B signaling regulates physiological and pathological chondrogenesis. *International Journal of Molecular Sciences*.

[43] Ni R., Song G., Fu X. (2020). Reactive oxygen species-responsive dexamethasone-loaded nanoparticles for targeted treatment of rheumatoid arthritis via suppressing the iRhom2/TNF-*α*/BAFF signaling pathway. *Biomaterials*.

[44] de Souza T. R., de Albuquerque Tavares Carvalho A., Duarte Â. P., Porter S. R., Leão J. C., Gueiros L. A. (2014). Th1 and Th2 polymorphisms in Sjögren's syndrome and rheumatoid arthritis. *Journal of Oral Pathology & Medicine*.

[45] Arleevskaya M. I., Larionova R. V., Brooks W. H., Bettacchioli E., Renaudineau Y. (2020). Toll-like receptors, infections, and rheumatoid arthritis. *Clinical Reviews in Allergy and Immunology*.

