# Supplementary material for: Computation-Based Discovery of Potential Targets for Rheumatoid Arthritis and Related Molecular Screening and Mechanism Analysis of Traditional Chinese Medicine
Source: Dis Markers. 2022 Jun 4;2022:1905077. doi: 10.1155/2022/1905077 (PMC9190478; doi:10.1155/2022/1905077)
Supplement: Supplementary 1 — Supporting material 1: information on 432 small molecules of TCM. [file 1905077.f1.doc]

| **Supporting material 1**: Information on 432 small molecules of TCM | | | |
| --- | --- | --- | --- |
| **Name** | **CAS** | **PubChem CID** | **Molecular Formula** |
| Fumaric acid | 110-17-8 | 444972 | C4H4O4 |
| Mannitol | 87-78-5 | 6251 | C6H14O6 |
| D-Galactose | 59-23-4 | 6036 | C6H12O6 |
| Succinic acid | 110-15-6 | 1110 | C4H6O4 |
| gamma-Aminobutyric acid | 56-12-2 | 119 | C4H9NO2 |
| Taurine | 107-35-7 | 1123 | C2H7NO3S |
| Saikosaponin A | 20736-09-8 | 167928 | C42H68O13 |
| Saikosaponin D | 20874-52-6 | 107793 | C42H68O13 |
| Betaine | 107-43-7 | 247 | C5H11NO2 |
| Sinapine | 18696-26-9 | 5280385 | C16H24NO5+ |
| Magnoflorine | 2141-09-5 | 73337 | C20H24NO4+ |
| Stachydrine | 471-87-4 | 115244 | C7H13NO2 |
| Stachydrine hydrochloride | 4136-37-2 | 44150282 | C7H14ClNO2 |
| Chelerythrine | 34316-15-9 | 2703 | C21H18NO4+ |
| Sanguinarine | 2447-54-3 | 5154 | C20H14NO4+ |
| Trigonelline | 535-83-1 | 5570 | C7H7NO2 |
| Fagaronine | 52259-65-1 | 40305 | C21H20NO4+ |
| Nitidine | 6872-57-7 | 4501 | C21H18NO4+ |
| Cynaropicrin | 35730-78-0 | 119093 | C19H22O6 |
| Dehydrocostus lactone | 477-43-0 | 73174 | C15H18O2 |
| Gentianine | 439-89-4 | 354616 | C10H9NO2 |
| Vernodalin | 21871-10-3 | 179375 | C19H20O7 |
| Swertiamarin | 17388-39-5 | 442435 | C16H22O10 |
| Gentiopicrin | 20831-76-9 | 88708 | C16H20O9 |
| Sweroside | 14215-86-2 | 161036 | C16H22O9 |
| Magnolol | 528-43-8 | 72300 | C18H18O2 |
| Honokiol | 35354-74-6 | 72303 | C18H18O2 |
| Allicin | 539-86-6 | 65036 | C6H10OS2 |
| Diallyl trisulfide | 2050-87-5 | 16315 | C6H10S3 |
| Allantoin | 97-59-6 | 204 | C4H6N4O3 |
| Gallic acid | 149-91-7 | 370 | C7H6O5 |
| Myricetin | 529-44-2 | 5281672 | C15H10O8 |
| 5-Hydroxymethylfurfural | 67-47-0 | 237332 | C6H6O3 |
| Ibotenic acid | 2552-55-8 | 1233 | C5H6N2O4 |
| Mangiferin | 4773-96-0 | 5281647 | C19H18O11 |
| Protocatechuic acid | 99-50-3 | 72 | C7H6O4 |
| Caffeic acid | 331-39-5 | 689043 | C9H8O4 |
| Protocatehuic aldehyde | 139-85-5 | 8768 | C7H6O3 |
| Quercetin | 117-39-5 | 5280343 | C15H10O7 |
| Hyperoside | 482-36-0 | 5281643 | C21H20O12 |
| Avicularin | 572-30-5 | 5490064 | C20H18O11 |
| Luteolin | 491-70-3 | 5280445 | C15H10O6 |
| Luteoloside | 5373-11-5 | 5280637 | C21H20O11 |
| Taxifolin | 480-18-2 | 439533 | C15H12O7 |
| Danshensu | 76822-21-4 | 11600642 | C9H10O5 |
| Salvianolic acid B | 115939-25-8 | 11629084 | C36H30O16 |
| Morin | 480-16-0 | 5281670 | C15H10O7 |
| Daphnetin | 486-35-1 | 5280569 | C9H6O4 |
| Hydroxysafflor Yellow A | 146087-19-6 | 91884958 | C27H32O16 |
| Carthamin | 36338-96-2 | 135565560 | C43H42O22 |
| Astragalus polyphenols | 55327-45-2 | 5321884 | C20H22O9 |
| Polydatin | 27208-80-6 | 5281718 | C20H22O8 |
| Kaempferol | 520-18-3 | 5280863 | C15H10O6 |
| Scutellarin | 27740-01-8 | 185617 | C21H18O12 |
| Apigenin | 520-36-5 | 5280443 | C15H10O5 |
| Vitexin | 3681-93-4 | 5280441 | C21H20O10 |
| Daidzein | 486-66-8 | 5281708 | C15H10O4 |
| Puerarin | 3681-99-0 | 5281807 | C21H20O9 |
| Genistein | 446-72-0 | 5280961 | C15H10O5 |
| Sophoricoside | 152-95-4 | 5321398 | C21H20O10 |
| Salidroside | 10338-51-9 | 159278 | C14H20O7 |
| Gastrodin | 62499-27-8 | 115067 | C13H18O7 |
| Arbutin | 497-76-7 | 440936 | C12H16O7 |
| Umbelliferone | 93-35-6 | 5281426 | C9H6O3 |
| Esculetin | 305-01-1 | 5281416 | C9H6O4 |
| Esculin | 531-75-9 | 5281417 | C15H16O9 |
| Psoralen | 66-97-7 | 6199 | C11H6O3 |
| Amygdalin | 29883-15-6 | 656516 | C20H27NO11 |
| Cinnamic acid | 621-82-9 | 444539 | C9H8O2 |
| Picroside I | 27409-30-9 | 6440892 | C24H28O11 |
| Cinnamaldehyde | 104-55-2 | 637511 | C9H8O |
| Galangin | 548-83-4 | 5281616 | C15H10O5 |
| Baicalin | 21967-41-9 | 64982 | C21H18O11 |
| Chrysin | 480-40-0 | 5281607 | C15H10O4 |
| Baicalein | 491-67-8 | 5281605 | C15H10O5 |
| Dicumarol | 66-76-2 | 54676038 | C19H12O6 |
| Indirubin | 479-41-4 | 10177 | C16H10N2O2 |
| Alizarin | 72-48-0 | 6293 | C14H8O4 |
| Rhein | 478-43-3 | 10168 | C15H8O6 |
| Aloeemodin | 481-72-1 | 10207 | C15H10O5 |
| Barbaloin | 1415-73-2 | 12305761 | C21H22O9 |
| Sennoside A | 81-27-6 | 73111 | C42H38O20 |
| Sennoside B | 128-57-4 | 91440 | C42H38O20 |
| Juglone | 481-39-0 | 3806 | C10H6O3 |
| Angelicin | 523-50-2 | 10658 | C11H6O3 |
| Catalpol | 2415-24-9 | 91520 | C15H22O10 |
| Adenosine | 58-61-7 | 60961 | C10H13N5O4 |
| Chlorogenic acid | 327-97-9 | 1794427 | C16H18O9 |
| Cianidanol | 154-23-4 | 9064 | C15H14O6 |
| Cordycepin | 73-03-0 | 6303 | C10H13N5O3 |
| Liquiritin | 551-15-5 | 503737 | C21H22O9 |
| Naringetol | 480-41-1 | 439246 | C15H12O5 |
| Brazilin | 474-07-7 | 73384 | C16H14O5 |
| Trifolirhizin | 6807-83-6 | 442827 | C22H22O10 |
| Sesamin | 607-80-7 | 72307 | C20H18O6 |
| Cytisine | 485-35-8 | 10235 | C11H14N2O |
| Oxymatrine | 16837-52-8 | 114850 | C15H24N2O2 |
| Matrine | 519-02-8 | 91466 | C15H24N2O |
| Sophocarpine | 6483-15-4 | 115269 | C15H22N2O |
| Piperine | 94-62-2 | 638024 | C17H19NO3 |
| Securinine | 5610-40-2 | 442872 | C13H15NO2 |
| Anabasine | 494-52-0 | 205586 | C10H14N2 |
| Rutaecarpine | 84-26-4 | 65752 | C18H13N3O |
| Lycorine | 476-28-8 | 72378 | C16H17NO4 |
| Strychnine | 57-24-9 | 441071 | C21H22N2O2 |
| Higenamine | 5843-65-2 | 114840 | C16H17NO3 |
| Tutin | 2571-22-4 | 75729 | C15H18O6 |
| Coriamyrtin | 2571-86-0 | 433737 | C15H18O5 |
| beta-Elemene | 515-13-9 | 6918391 | C15H24 |
| Betulonic acid | 4481-62-3 | 122844 | C30H46O3 |
| Betulinic acid | 472-15-1 | 64971 | C30H48O3 |
| Betulin | 473-98-3 | 72326 | C30H50O2 |
| Crocetin | 27876-94-4 | 5281232 | C20H24O4 |
| Crocin II | 55750-84-0 | 9940690 | C38H54O19 |
| alpha-Crocin | 42553-65-1 | 5281233 | C44H64O24 |
| Pseudolaric acid B | 82508-31-4 | 71307573 | C23H28O8 |
| Alkannin | 517-88-4 | 72521 | C16H16O5 |
| Acetylshikonin | 24502-78-1 | 479501 | C18H18O6 |
| beta,beta-Dimethylacrylshikonin | 24502-79-2 | 479499 | C21H22O6 |
| Isobavachalcone | 20784-50-3 | 5281255 | C20H20O4 |
| Osthole | 484-12-8 | 10228 | C15H16O3 |
| Lapachol | 84-79-7 | 3884 | C15H14O3 |
| Citral | 5392-40-5 | 638011 | C10H16O |
| Geraniol | 106-24-1 | 637566 | C10H18O |
| Bakuchiol | 10309-37-2 | 5468522 | C18H24O |
| Ginsenoside Rg1 | 22427-39-0 | 441923 | C42H72O14 |
| Ginsenoside Rf | 52286-58-5 | 441922 | C42H72O14 |
| Notoginsenoside R1 | 80418-24-2 | 441934 | C47H80O18 |
| Ginsenoside Rb1 | 41753-43-9 | 9898279 | C54H92O23 |
| Ginsenoside Rb2 | 11021-13-9 | 6917976 | C53H90O22 |
| Ginsenoside Rc | 11021-14-0 | 12855889 | C53H90O22 |
| Gambogic acid | 2752-65-0 | 9852185 | C38H44O8 |
| Imperatorin | 482-44-0 | 10212 | C16H14O4 |
| Isoimperatorin | 482-45-1 | 68081 | C16H14O4 |
| Paeonol | 552-41-0 | 11092 | C9H10O3 |
| Colchicine | 64-86-8 | 6167 | C22H25NO6 |
| Cucurbitacin E | 18444-66-1 | 5281319 | C32H44O8 |
| Cucurbitacin B | 6199-67-3 | 5281316 | C32H46O8 |
| Cinobufagin | 470-37-1 | 11969542 | C26H34O6 |
| Toosendanin | 58812-37-6 | 9851101 | C30H38O11 |
| (-)-Bornyl acetate | 76-49-3 | 93009 | C12H20O2 |
| Ephedrine | 299-42-3 | 9294 | C10H15NO |
| Ephedrine hydrochloride | 50-98-6 | 65326 | C10H16ClNO |
| Bilobalide | 33570-04-6 | 73581 | C15H18O8 |
| Cimifugin | 37921-38-3 | 441960 | C16H18O6 |
| Prim-O-glucosylcimifugin | 80681-45-4 | 14034912 | C22H28O11 |
| Harringtonine | 26833-85-2 | 276389 | C28H37NO9 |
| Homoharringtonine | 26833-87-4 | 285033 | C29H39NO9 |
| Capsaicin | 404-86-4 | 1548943 | C18H27NO3 |
| Nagilactone C | 24338-53-2 | 72505 | C19H22O7 |
| Tripdiolide | 38647-10-8 | 294491 | C20H24O7 |
| Triptonide | 38647-11-9 | 65411 | C20H22O6 |
| Triptolide | 38748-32-2 | 107985 | C20H24O6 |
| Lupulone | 468-28-0 | 68051 | C26H38O4 |
| Humulone | 26472-41-3 | 442911 | C21H30O5 |
| Cyclovirobuxine D | 860-79-7 | 260439 | C26H46N2O |
| Baccharin | 61251-97-6 | 5358645 | C29H38O11 |
| Alisol A 24-acetate | 18674-16-3 | 76336194 | C32H52O6 |
| Sodium taurocholate | 145-42-6 | 23666345 | C26H44NNaO7S |
| Cholic acid | 81-25-4 | 221493 | C24H40O5 |
| Deoxycholic acid | 83-44-3 | 222528 | C24H40O4 |
| Chenodeoxycholic acid | 474-25-9 | 10133 | C24H40O4 |
| Ergonovine | 60-79-7 | 443884 | C19H23N3O2 |
| Aescine | 6805-41-0 | 16211024 | C55H86O24 |
| Praeruptorin A | 73069-25-7 | 38347601 | C21H22O7 |
| Fucosterol | 17605-67-3 | 5281326 | C29H48O |
| Oleuropein | 32619-42-4 | 5281544 | C25H32O13 |
| Huperzine A | 102518-79-6 | 449069 | C15H18N2O |
| Lobetyolin | 136085-37-5 | 53486204 | C20H28O8 |
| alpha-Asarone | 2883-98-9 | 636822 | C12H16O3 |
| Anethole | 104-46-1 | 637563 | C10H12O |
| Ergotamine | 113-15-5 | 8223 | C33H35N5O5 |
| Astragaloside IV | 84687-43-4 | 13943297 | C41H68O14 |
| Oxypeucedanin | 737-52-0 | 160544 | C16H14O5 |
| Mollugin | 55481-88-4 | 124219 | C17H16O4 |
| Cucurbitacin I | 2222-07-3 | 5281321 | C30H42O7 |
| Cucurbitacin D | 3877-86-9 | 5281318 | C30H44O7 |
| Limonin | 1180-71-8 | 179651 | C26H30O8 |
| Miroestrol | 2618-41-9 | 165001 | C20H22O6 |
| Eucalyptol | 470-82-6 | 2758 | C10H18O |
| D-Camphor | 464-49-3 | 159055 | C10H16O |
| (-)-Isoborneol | 507-70-0 | 6321405 | C10H18O |
| 3-Bornanol | 1686-28-8 | 565679 | C10H18O |
| Glycyrrhetic acid | 471-53-4 | 10114 | C30H46O4 |
| Glycyrrhizic acid | 1405-86-3 | 14982 | C42H62O16 |
| Harpagide | 6926-08-5 | 10044294 | C15H24O10 |
| Harpagoside | 19210-12-9 | 5281542 | C24H30O11 |
| Oridonin | 28957-04-2 | 5321010 | C20H28O6 |
| Ponicidin | 52617-37-5 | 92043456 | C20H26O6 |
| Polygalacic acid | 22338-71-2 | 161388 | C30H48O6 |
| Oleanolic acid | 508-02-1 | 10494 | C30H48O3 |
| Hederagenin | 465-99-6 | 73299 | C30H48O4 |
| Kirenol | 52659-56-0 | 15736732 | C20H34O4 |
| Psorospermin | 74045-97-9 | 126451 | C19H16O6 |
| Farrerol | 24211-30-1 | 91144 | C17H16O5 |
| Celastrol | 34157-83-0 | 122724 | C29H38O4 |
| Pristimerin | 1258-84-0 | 159516 | C30H40O4 |
| Maltol | 118-71-8 | 8369 | C6H6O3 |
| Bruceantin | 41451-75-6 | 5281304 | C28H36O11 |
| Brusatol | 14907-98-3 | 73432 | C26H32O11 |
| Withaferin A | 5119-48-2 | 265237 | C28H38O6 |
| Carvacrol | 499-75-2 | 10364 | C10H14O |
| Pyrolin | 95-71-6 | 7253 | C7H8O2 |
| Dracorhodin | 643-56-1 | 69509 | C17H14O3 |
| Ligustrazine | 1124-11-4 | 14296 | C8H12N2 |
| Bilirubin | 635-65-4 | 5280352 | C33H36N4O6 |
| Fraxinellone | 28808-62-0 | 124039 | C14H16O3 |
| Paclitaxel | 33069-62-4 | 36314 | C47H51NO14 |
| Lactucin | 1891-29-8 | 442266 | C15H16O5 |
| Atractylenolide I | 73069-13-3 | 5321018 | C15H18O2 |
| Thymol | 89-83-8 | 6989 | C10H14O |
| Bruceine D | 21499-66-1 | 71463728 | C20H26O9 |
| Plumbagin | 481-42-5 | 10205 | C11H8O3 |
| Gossypol | 303-45-7 | 3503 | C30H30O8 |
| Emodin | 518-82-1 | 3220 | C15H10O5 |
| Physcion | 521-61-9 | 10639 | C16H12O5 |
| Chrysophanol | 481-74-3 | 10208 | C15H10O4 |
| Eupatolide | 6750-25-0 | 10399772 | C15H20O3 |
| Deoxyelephantopin | 29307-03-7 | 99904 | C19H20O6 |
| Germacrone | 6902-91-6 | 6436348 | C15H22O |
| alpha-Bisabolol | 515-69-5 | 10586 | C15H26O |
| Cnicin | 24394-09-0 | 5281435 | C20H26O7 |
| Costunolide | 553-21-9 | 5281437 | C15H20O2 |
| Lipiferolide | 41059-80-7 | 54598331 | C17H22O5 |
| alpha-Eudesmol | 473-16-5 | 92762 | C15H26O |
| Linderane | 13476-25-0 | 6915739 | C15H16O4 |
| Tanshinone IIA | 568-72-9 | 164676 | C19H18O3 |
| Harmine | 442-51-3 | 5280953 | C13H12N2O |
| Harmalol | 525-57-5 | 3565 | C12H12N2O |
| Harmaline | 304-21-2 | 3564 | C13H14N2O |
| Corynoline | 18797-79-0 | 177014 | C21H21NO5 |
| Cantharidin | 56-25-7 | 5944 | C10H12O4 |
| Paeoniflorin | 23180-57-6 | 442534 | C23H28O11 |
| Andrographolide | 5508-58-7 | 5318517 | C20H30O5 |
| Resibufogenin | 465-39-4 | 6917974 | C24H32O4 |
| Strophanthidin | 66-28-4 | 6185 | C23H32O6 |
| Estriol | 50-27-1 | 5756 | C18H24O3 |
| Estradiol | 50-28-2 | 5757 | C18H24O2 |
| Isoalantolactone | 470-17-7 | 73285 | C15H20O2 |
| Friedelin | 559-74-0 | 91472 | C30H50O |
| Ginkgolide A | 15291-75-5 | 115221 | C20H24O9 |
| Ginkgolide B | 15291-77-7 | 65243 | C20H24O10 |
| Ginkgolide C | 15291-76-6 | 24721502 | C20H24O11 |
| Monocrotaline | 315-22-0 | 9415 | C16H23NO6 |
| Icariin | 489-32-7 | 5318997 | C33H40O15 |
| Quercitrin | 522-12-3 | 5280459 | C21H20O11 |
| Kaempferitrin | 482-38-2 | 5486199 | C27H30O14 |
| Neohesperidin | 13241-33-3 | 442439 | C28H34O15 |
| Naringin | 10236-47-2 | 442428 | C27H32O14 |
| Raddeanin A | 89412-79-3 | 174742 | C47H76O16 |
| Ginsenoside Re | 52286-59-6 | 441921 | C48H82O18 |
| Tubeimoside I | 102040-03-9 | 51346132 | C63H98O29 |
| Platycodin D | 58479-68-8 | 162859 | C57H92O28 |
| Jujuboside B | 55466-05-2 | 24721031 | C52H84O21 |
| Jujuboside A | 55466-04-1 | 51346169 | C58H94O26 |
| Verbascoside | 61276-17-3 | 5281800 | C29H36O15 |
| Echinacoside | 82854-37-3 | 5281771 | C35H46O20 |
| Astilbin | 29838-67-3 | 119258 | C21H22O11 |
| Buddlejasaponin IV | 139523-30-1 | 153940 | C48H78O18 |
| Pectolinarin | 28978-02-1 | 168849 | C29H34O15 |
| Rutin | 153-18-4 | 5280805 | C27H30O16 |
| Typhaneoside | 104472-68-6 | 5489389 | C34H42O20 |
| Robinin | 301-19-9 | 5281693 | C33H40O19 |
| Linarin | 480-36-4 | 5317025 | C28H32O14 |
| Hesperidin | 520-26-3 | 10621 | C28H34O15 |
| Digoxin | 20830-75-5 | 2724385 | C41H64O14 |
| Divaricoside | 508-84-9 | 120704 | C30H46O8 |
| Loganin | 18524-94-2 | 87691 | C17H26O10 |
| Friedelanol | 5085-72-3 | 101341 | C30H52O |
| Alstonine | 642-18-2 | 170780 | C21H21N2O3+ |
| Maytansine | 35846-53-8 | 5281828 | C34H46ClN3O10 |
| Conessine | 546-06-5 | 441082 | C24H40N2 |
| Calotropin | 1986-70-5 | 16142 | C29H40O9 |
| Schisandrin | 7432-28-2 | 23915 | C24H32O7 |
| Schizandrin A | 61281-38-7 | 155256 | C24H32O6 |
| Schisantherin A | 58546-56-8 | 151529 | C30H32O9 |
| Schisandrin B | 61281-37-6 | 108130 | C23H28O6 |
| Jatrophon | 29444-03-9 | 5281373 | C20H24O3 |
| Menthol | 1490-04-6 | 1254 | C10H20O |
| Curdione | 13657-68-6 | 6441391 | C15H24O2 |
| Polyphyllin VI | 55916-51-3 | 10417550 | C39H62O13 |
| Solasodine | 126-17-0 | 442985 | C27H43NO2 |
| Diosgenin | 512-04-9 | 99474 | C27H42O3 |
| Dioscin | 19057-60-4 | 119245 | C45H72O16 |
| Polyphyllin I | 50773-41-6 | 11018329 | C44H70O16 |
| Polyphyllin II | 76296-72-5 | 46200821 | C44H70O16 |
| Sarsasapogenin | 126-19-2 | 92095 | C27H44O3 |
| Tomatine | 17406-45-0 | 28523 | C50H83NO21 |
| Asiaticoside | 16830-15-2 | 24721205 | C48H78O19 |
| Ursolic acid | 77-52-1 | 64945 | C30H48O3 |
| Artemisinin | 63968-64-9 | 68827 | C15H22O5 |
| Ambrosin | 509-93-3 | 92119 | C15H18O3 |
| Cevane-3,6,14,16,20-pentol | 82841-67-6 | 158259 | C27H45NO5 |
| alpha-Solanin | 20562-02-1 | 9549171 | C45H73NO15 |
| Imperialine | 61825-98-7 | 442977 | C27H43NO3 |
| Peimine | 23496-41-5 | 131900 | C27H45NO3 |
| Hupehenine | 98243-57-3 | 14240934 | C27H45NO2 |
| Curcumenol | 19431-84-6 | 167812 | C15H22O2 |
| Curcumol | 4871-97-0 | 14240392 | C15H24O2 |
| Alantolactone | 546-43-0 | 72724 | C15H20O2 |
| Gnidimacrin | 60796-70-5 | 3085204 | C44H54O12 |
| Muscone | 541-91-3 | 10947 | C16H30O |
| Simalikilactone D | 35321-80-3 | 441808 | C25H34O9 |
| Trilobolide | 50657-07-3 | 5281503 | C27H38O10 |
| Hirudin | 113274-56-9 | 16138839 | C66H93N13O25 |
| beta-Sitosterol | 83-46-5 | 222284 | C29H50O |
| 10-Hydroxycamptothecin | 19685-09-7 | 97226 | C20H16N2O5 |
| Camptothecine | 7689-03-4 | 24360 | C20H16N2O4 |
| Vincaleukoblastine | 865-21-4 | 241903 | C46H58N4O9 |
| Vincristine | 57-22-7 | 5978 | C46H56N4O10 |
| Vincamine | 1617-90-9 | 15376 | C21H26N2O3 |
| Voacanginine | 3371-85-5 | 11953931 | C43H52N4O5 |
| Tabernaemontanin | 2134-98-7 | 12309360 | C21H26N2O3 |
| Rhynchophylline | 76-66-4 | 5281408 | C22H28N2O4 |
| Emetine | 483-18-1 | 10219 | C29H40N2O4 |
| Agrimophol | 65792-05-4 | 442901 | C26H34O8 |
| 6-Gingerol | 23513-14-6 | 442793 | C17H26O4 |
| Trilinolein | 537-40-6 | 5322095 | C57H98O6 |
| Ginkgolic acid | 22910-60-7 | 5281858 | C22H34O3 |
| Coixenolide | 29066-43-1 | 46173943 | C38H70O4 |
| 2-Undecanone | 112-12-9 | 8163 | C11H22O |
| Houttuynin | 56505-80-7 | 122640 | C12H22O2 |
| Embelin | 550-24-3 | 3218 | C17H26O4 |
| Ginkgoneolic acid | 20261-38-5 | 161306 | C20H32O3 |
| Aconitine | 302-27-2 | 245005 | C34H47NO11 |
| Songorine | 509-24-0 | 71456946 | C22H31NO3 |
| Arecoline | 18513-76-3 | 205701 | C8H13NO2 |
| Gramine | 87-52-5 | 6890 | C11H14N2 |
| Thaspine | 602-07-3 | 215159 | C20H19NO6 |
| Thaspine hydrochloride | 35115-50-5 | 215158 | C20H20ClNO6 |
| Lobeline | 90-69-7 | 101616 | C22H27NO2 |
| Caffeine | 58-08-2 | 2519 | C8H10N4O2 |
| Theophylline | 58-55-9 | 2153 | C7H8N4O2 |
| Evodiamine | 518-17-2 | 442088 | C19H17N3O |
| Anisodamine | 55869-99-3 | 2198 | C17H23NO4 |
| Anisodine | 52646-92-1 | 11616712 | C17H21NO5 |
| Scopolamine | 51-34-3 | 3000322 | C17H21NO4 |
| Scopolamine hydrobromide | 114-49-8 | 6603108 | C17H22BrNO4 |
| Atropine | 51-55-8 | 174174 | C17H23NO3 |
| Atropine sulphate | 55-48-1 | 60196398 | C34H48N2O10S |
| Gelsemine | 509-15-9 | 5390854 | C20H22N2O2 |
| Chelidonine | 476-32-4 | 197810 | C20H19NO5 |
| Arecoline | 63-75-2 | 2230 | C8H13NO2 |
| Liensinine | 2586-96-1 | 160644 | C37H42N2O6 |
| Neferine | 2292-16-2 | 159654 | C38H44N2O6 |
| Dauricine | 524-17-4 | 73400 | C38H44N2O6 |
| Pronuciferine | 2128-60-1 | 200480 | C19H21NO3 |
| Fangchinoline | 436-77-1 | 73481 | C37H40N2O6 |
| Berbamine | 478-61-5 | 275182 | C37H40N2O6 |
| Isotetrandrine | 477-57-6 | 457825 | C38H42N2O6 |
| Tubocurarine | 57-95-4 | 6000 | C37H41N2O6+ |
| Curine | 436-05-5 | 253793 | C36H38N2O6 |
| Protopine | 130-86-9 | 4970 | C20H19NO5 |
| Stephanine | 517-63-5 | 160501 | C19H19NO3 |
| Cepharanthine | 481-49-2 | 10206 | C37H38N2O6 |
| Galantamine | 357-70-0 | 9651 | C17H21NO3 |
| Morphine | 57-27-2 | 5288826 | C17H19NO3 |
| Codeine phosphate | 52-28-8 | 5359227 | C18H24NO7P |
| Sinomenine | 115-53-7 | 5459308 | C19H23NO4 |
| Abrine | 526-31-8 | 160511 | C12H14N2O2 |
| Colchamine | 477-30-5 | 220401 | C21H25NO5 |
| Synephrine | 94-07-5 | 7172 | C9H13NO2 |
| Methyl salicylate | 119-36-8 | 4133 | C8H8O3 |
| Genipin | 6902-77-8 | 442424 | C11H14O5 |
| Geniposide | 24512-63-8 | 107848 | C17H24O10 |
| Jacaranone | 60263-07-2 | 73307 | C9H10O4 |
| Fraxetin | 574-84-5 | 5273569 | C10H8O5 |
| Isofraxidin | 486-21-5 | 5318565 | C11H10O5 |
| Curculigoside | 85643-19-2 | 158845 | C22H26O11 |
| Isoferulic acid | 537-73-5 | 736186 | C10H10O4 |
| Rhapontin | 155-58-8 | 637213 | C21H24O9 |
| Vitexicarpin | 479-91-4 | 5315263 | C19H18O8 |
| Eupatorin | 855-96-9 | 97214 | C18H16O7 |
| Nobiletin | 478-01-3 | 72344 | C21H22O8 |
| Eupatilin | 22368-21-4 | 5273755 | C18H16O7 |
| Forsythoside D | 487-41-2 | 24721571 | C27H34O11 |
| Magnolin | 31008-18-1 | 169234 | C23H28O7 |
| Phillyrin | 487-41-2 | 101712 | C27H34O11 |
| Methyleugenol | 93-15-2 | 7127 | C11H14O2 |
| Papaverine | 58-74-2 | 4680 | C20H21NO4 |
| Arctigenin | 7770-78-7 | 64981 | C21H24O6 |
| Arctiin | 20362-31-6 | 100528 | C27H34O11 |
| Wogonin | 632-85-9 | 5281703 | C16H12O5 |
| Daphnoretin | 2034-69-7 | 5281406 | C19H12O7 |
| Scopoletin | 92-61-5 | 5280460 | C10H8O4 |
| Scoparone | 120-08-1 | 8417 | C11H10O4 |
| Scopolin | 531-44-2 | 439514 | C16H18O9 |
| Tylophorine | 482-20-2 | 92114 | C24H27NO4 |
| Brucine | 357-57-3 | 442021 | C23H26N2O4 |
| Tectoridin | 611-40-5 | 5281810 | C22H22O11 |
| Bergenin | 477-90-7 | 66065 | C14H16O9 |
| Vanillic acid | 121-34-6 | 8468 | C8H8O4 |
| Picroside II | 39012-20-9 | 3081484 | C23H28O13 |
| Curcumin | 458-37-7 | 969516 | C21H20O6 |
| Ferulic acid | 1135-24-6 | 445858 | C10H10O4 |
| Isorhamnetin | 480-19-3 | 5281654 | C16H12O7 |
| Silibinin | 22888-70-6 | 31553 | C25H22O10 |
| Pinoresinol | 487-36-5 | 73399 | C20H22O6 |
| Eugenol | 97-53-0 | 3314 | C10H12O2 |
| Vanillyl alcohol | 498-00-0 | 62348 | C8H10O3 |
| Eupafolin | 520-11-6 | 5317284 | C16H12O7 |
| Hispidulin | 1447-88-7 | 5281628 | C16H12O6 |
| Tectorigenin | 548-77-6 | 5281811 | C16H12O6 |
| Tetrahydropalmatine | 10097-84-4 | 5417 | C21H25NO4 |
| Tetrahydropalmatine | 483-14-7 | 72301 | C21H25NO4 |
| Jatrorrhizine | 3621-38-3 | 72323 | C20H20NO4+ |
| Palmatine | 3486-67-7 | 19009 | C21H22NO4+ |
| Berberine | 2086-83-1 | 2353 | C20H18NO4+ |
| Berberine hydrochloride | 633-65-8 | 12456 | C20H18ClNO4 |
| Oxoglaucine | 5574-24-3 | 97662 | C20H17NO5 |
| Xanthotoxine | 298-81-7 | 4114 | C12H8O4 |
| Bergapten | 484-20-8 | 2355 | C12H8O4 |
| 4-Methoxysalicylaldehyde | 673-22-3 | 69600 | C8H8O3 |
| Cardamonin | 19309-14-9 | 641785 | C16H14O4 |
| Leonurine | 24697-74-3 | 161464 | C14H21N3O5 |
| Irisflorentin | 41743-73-1 | 170569 | C20H18O8 |
| Deoxypodophyllotoxin | 19186-35-7 | 345501 | C22H22O7 |
| Podophyllotoxin | 518-28-5 | 10607 | C22H22O8 |
| Podophyllotoxin glucoside | 16481-54-2 | 161177 | C28H32O13 |
| Syringin | 118-34-3 | 5316860 | C17H24O9 |
| Alpinetin | 36052-37-6 | 154279 | C16H14O4 |
| Acacetin | 480-44-4 | 5280442 | C16H12O5 |
| Formononetin | 485-72-3 | 5280378 | C16H12O4 |
| Biochanin A | 491-80-5 | 5280373 | C16H12O5 |
| Agrimonolide | 21499-24-1 | 161362 | C18H18O5 |
| Tylophorinidine | 32523-69-6 | 161749 | C22H23NO4 |
| Homopterocarpin | 606-91-7 | 101795 | C17H16O4 |
| Limettin | 487-06-9 | 2775 | C11H10O4 |
| Quinidine | 56-54-2 | 441074 | C20H24N2O2 |
| Quinine | 130-95-0 | 3034034 | C20H24O2N2 |
| Cephalotaxine | 24316-19-6 | 65305 | C18H21NO4 |
| Sulforaphane | 4478-93-7 | 5350 | C6H11NOS2 |
