# Supplementary material for: Computation-Based Discovery of Potential Targets for Rheumatoid Arthritis and Related Molecular Screening and Mechanism Analysis of Traditional Chinese Medicine
Source: Dis Markers. 2022 Jun 4;2022:1905077. doi: 10.1155/2022/1905077 (PMC9190478; doi:10.1155/2022/1905077)
Supplement: Supplementary 2 — Supporting material 2: ADMET evaluation results of 432 small molecules of TCM. [file 1905077.f2.doc]

| **Supporting material 2**: ADMET evaluation results of 432 small molecules of TCM | | | | | | | | | | | | |
| --- | --- | --- | --- | --- | --- | --- | --- | --- | --- | --- | --- | --- |
| **Name** | **Lipinski** | **MW** | **LogP** | **Solubility** | **BBB permeant** | **Pgp substrate** | **GI absorption** | **Bioavailability Score** | **Synthetic Accessibility** | **Metabolic Stability** | **Ames** | **hERG** |
| Fumaric acid | Good | 116.07 | Optimal | Soluble | No | No | High | 0.85 | 1.8 | Undefined | Non-mutagenic | Non-inhibitor |
| Mannitol | Bad | 182.17 | Very hydrophilic | Soluble | No | No | Low | 0.55 | 3.3 | Undefined | Non-mutagenic | Non-inhibitor |
| D-Galactose | Moderate | 180.16 | Very hydrophilic | Soluble | No | Yes | Low | 0.55 | 4.08 | Undefined | Non-mutagenic | Non-inhibitor |
| Succinic acid | Good | 118.09 | Optimal | Soluble | No | No | High | 0.85 | 1.29 | Stable in HLM | Non-mutagenic | Non-inhibitor |
| gamma-Aminobutyric acid | Good | 103.12 | Optimal | Soluble | No | No | High | 0.55 | 1 | Undefined | Non-mutagenic | Non-inhibitor |
| Taurine | Moderate | 125.15 | Very hydrophilic | Soluble | No | No | High | 0.55 | 2.17 | Undefined | Non-mutagenic | Undefined |
| Saikosaponin A | Bad | 780.98 | Optimal | Insoluble | No | Yes | Low | 0.17 | 9.86 | Undefined | Non-mutagenic | Non-inhibitor |
| Saikosaponin D | Bad | 780.98 | Optimal | Insoluble | No | Yes | Low | 0.17 | 9.86 | Undefined | Non-mutagenic | Non-inhibitor |
| Betaine | Moderate | 117.15 | Very hydrophilic | Soluble | No | No | Low | 0.55 | 1 | Undefined | Non-mutagenic | Non-inhibitor |
| Sinapine | Moderate | 310.37 | Very hydrophilic | Soluble | Yes | Yes | High | 0.55 | 2.9 | Undefined | Undefined | Undefined |
| Magnoflorine | Good | 342.41 | Hydrophilic | Soluble | Yes | Yes | High | 0.55 | 3.78 | Undefined | Undefined | Undefined |
| Stachydrine | Moderate | 143.18 | Very hydrophilic | Soluble | No | No | Low | 0.55 | 1.72 | Undefined | Non-mutagenic | Non-inhibitor |
| Stachydrine hydrochloride | Moderate | 179.64 | Very hydrophilic | Soluble | No | No | Low | 0.85 | 1.81 | Undefined | Non-mutagenic | Non-inhibitor |
| Chelerythrine | Good | 348.37 | Hydrophilic | Soluble | Yes | Yes | High | 0.55 | 2.77 | Undefined | Mutagenic | Inhibitor |
| Sanguinarine | Good | 332.33 | Hydrophilic | Soluble | Yes | Yes | High | 0.55 | 2.59 | Undefined | Undefined | Undefined |
| Trigonelline | Moderate | 137.14 | Very hydrophilic | Soluble | No | No | High | 0.55 | 1.04 | Undefined | Non-mutagenic | Non-inhibitor |
| Fagaronine | Good | 350.39 | Optimal | Highly insoluble | Yes | Yes | High | 0.55 | 2.09 | Undefined | Undefined | Undefined |
| Nitidine | Good | 348.37 | Hydrophilic | Soluble | Yes | Yes | High | 0.55 | 2.65 | Undefined | Mutagenic | Inhibitor |
| Cynaropicrin | Good | 346.37 | Optimal | Soluble | No | No | High | 0.55 | 4.7 | Undefined | Undefined | Undefined |
| Dehydrocostus lactone | Good | 230.3 | Optimal | Insoluble | Yes | No | High | 0.55 | 3.84 | Undefined | Non-mutagenic | Undefined |
| Gentianine | Good | 175.18 | Optimal | Soluble | Yes | No | High | 0.55 | 2.24 | Undefined | Undefined | Undefined |
| Vernodalin | Good | 360.36 | Optimal | Insoluble | No | No | High | 0.55 | 4.63 | Undefined | Undefined | Non-inhibitor |
| Swertiamarin | Moderate | 374.34 | Very hydrophilic | Soluble | No | No | Low | 0.11 | 5.56 | Undefined | Undefined | Non-inhibitor |
| Gentiopicrin | Moderate | 356.32 | Very hydrophilic | Soluble | No | Yes | Low | 0.56 | 5.55 | Undefined | Undefined | Non-inhibitor |
| Sweroside | Good | 358.34 | Hydrophilic | Soluble | No | Yes | Low | 0.56 | 5.5 | Undefined | Undefined | Non-inhibitor |
| Magnolol | Good | 266.33 | Optimal | Insoluble | Yes | No | High | 0.55 | 2.49 | Undefined | Non-mutagenic | Undefined |
| Honokiol | Good | 266.33 | Optimal | Insoluble | Yes | No | High | 0.55 | 2.45 | Undefined | Non-mutagenic | Undefined |
| Allicin | Good | 162.27 | Optimal | Soluble | Yes | No | High | 0.55 | 3.6 | Undefined | Undefined | Undefined |
| Diallyl trisulfide | Good | 178.34 | Optimal | Soluble | Yes | No | High | 0.55 | 3.58 | Undefined | Undefined | Undefined |
| Allantoin | Moderate | 158.12 | Very hydrophilic | Soluble | No | No | Low | 0.55 | 2.42 | Undefined | Undefined | Non-inhibitor |
| Gallic acid | Good | 170.12 | Optimal | Soluble | No | No | High | 0.56 | 1.22 | Undefined | Non-mutagenic | Non-inhibitor |
| Myricetin | Moderate | 318.24 | Optimal | Soluble | No | No | Low | 0.55 | 3.27 | Undefined | Mutagenic | Non-inhibitor |
| 5-Hydroxymethylfurfural | Good | 126.11 | Optimal | Soluble | No | No | High | 0.55 | 2.25 | Undefined | Undefined | Non-inhibitor |
| Ibotenic acid | Good | 158.11 | Optimal | Soluble | No | No | High | 0.55 | 2.86 | Undefined | Undefined | Non-inhibitor |
| Mangiferin | Bad | 422.34 | Optimal | Soluble | No | No | Low | 0.17 | 4.76 | Stable in HLM | Undefined | Non-inhibitor |
| Protocatechuic acid | Good | 154.12 | Optimal | Soluble | No | No | High | 0.56 | 1.07 | Undefined | Non-mutagenic | Non-inhibitor |
| Caffeic acid | Good | 180.16 | Optimal | Soluble | No | No | High | 0.56 | 1.81 | Undefined | Non-mutagenic | Non-inhibitor |
| Protocatehuic aldehyde | Good | 138.12 | Optimal | Soluble | Yes | No | High | 0.55 | 1 | Undefined | Non-mutagenic | Non-inhibitor |
| Quercetin | Good | 302.24 | Optimal | Soluble | No | No | High | 0.55 | 3.23 | Undefined | Undefined | Non-inhibitor |
| Hyperoside | Bad | 464.38 | Optimal | Soluble | No | No | Low | 0.17 | 5.32 | Undefined | Mutagenic | Non-inhibitor |
| Avicularin | Bad | 434.35 | Optimal | Soluble | No | No | Low | 0.17 | 5.04 | Undefined | Mutagenic | Non-inhibitor |
| Luteolin | Good | 286.24 | Optimal | Soluble | No | No | High | 0.55 | 3.02 | Undefined | Undefined | Non-inhibitor |
| Luteoloside | Bad | 448.38 | Optimal | Soluble | No | Yes | Low | 0.17 | 5.17 | Undefined | Undefined | Non-inhibitor |
| Taxifolin | Good | 304.25 | Optimal | Soluble | No | No | High | 0.55 | 3.51 | Stable in HLM | Undefined | Non-inhibitor |
| Danshensu | Good | 198.17 | Optimal | Soluble | No | No | High | 0.56 | 1.91 | Undefined | Non-mutagenic | Undefined |
| Salvianolic acid B | Bad | 718.61 | Optimal | Soluble | No | No | Low | 0.11 | 6 | Undefined | Undefined | Non-inhibitor |
| Morin | Good | 302.24 | Optimal | Soluble | No | No | High | 0.55 | 3.25 | Undefined | Mutagenic | Non-inhibitor |
| Daphnetin | Good | 178.14 | Optimal | Soluble | No | No | High | 0.55 | 2.79 | Undefined | Undefined | Non-inhibitor |
| Hydroxysafflor Yellow A | Bad | 612.53 | Optimal | Soluble | No | No | Low | 0.11 | 6.74 | Undefined | Undefined | Non-inhibitor |
| Carthamin | Bad | 910.78 | Optimal | Soluble | No | Yes | Low | 0.11 | 8.15 | Undefined | Undefined | Non-inhibitor |
| Astragalus polyphenols | Moderate | 406.38 | Optimal | Soluble | No | Yes | Low | 0.55 | 4.96 | Undefined | Undefined | Non-inhibitor |
| Polydatin | Moderate | 390.38 | Optimal | Soluble | No | Yes | High | 0.55 | 4.82 | Undefined | Undefined | Undefined |
| Kaempferol | Good | 286.24 | Optimal | Soluble | No | No | High | 0.55 | 3.14 | Undefined | Mutagenic | Non-inhibitor |
| Scutellarin | Bad | 462.36 | Optimal | Soluble | No | Yes | Low | 0.11 | 5.12 | Undefined | Undefined | Non-inhibitor |
| Apigenin | Good | 270.24 | Optimal | Soluble | No | No | High | 0.55 | 2.96 | Undefined | Undefined | Non-inhibitor |
| Vitexin | Moderate | 432.38 | Optimal | Soluble | No | No | Low | 0.55 | 5.12 | Undefined | Undefined | Non-inhibitor |
| Daidzein | Good | 254.24 | Optimal | Insoluble | Yes | No | High | 0.55 | 2.79 | Undefined | Undefined | Non-inhibitor |
| Puerarin | Moderate | 416.38 | Optimal | Soluble | No | No | Low | 0.55 | 4.98 | Undefined | Undefined | Non-inhibitor |
| Genistein | Good | 270.24 | Optimal | Soluble | No | No | High | 0.55 | 2.87 | Undefined | Undefined | Non-inhibitor |
| Sophoricoside | Moderate | 432.38 | Optimal | Soluble | No | Yes | Low | 0.55 | 5 | Undefined | Undefined | Non-inhibitor |
| Salidroside | Good | 300.3 | Optimal | Soluble | No | No | High | 0.55 | 4.26 | Stable in HLM | Undefined | Non-inhibitor |
| Gastrodin | Good | 286.28 | Hydrophilic | Soluble | No | No | Low | 0.55 | 4.1 | Undefined | Undefined | Non-inhibitor |
| Arbutin | Good | 272.25 | Hydrophilic | Soluble | No | No | High | 0.55 | 4.18 | Undefined | Non-mutagenic | Non-inhibitor |
| Umbelliferone | Good | 162.14 | Optimal | Soluble | Yes | No | High | 0.55 | 2.56 | Undefined | Undefined | Non-inhibitor |
| Esculetin | Good | 178.14 | Optimal | Soluble | No | No | High | 0.55 | 2.61 | Undefined | Undefined | Non-inhibitor |
| Esculin | Good | 340.28 | Hydrophilic | Soluble | No | No | Low | 0.55 | 4.69 | Undefined | Undefined | Non-inhibitor |
| Psoralen | Good | 186.16 | Optimal | Soluble | Yes | No | High | 0.55 | 3.06 | Undefined | Undefined | Undefined |
| Amygdalin | Bad | 457.43 | Hydrophilic | Soluble | No | No | Low | 0.17 | 5.41 | Undefined | Undefined | Non-inhibitor |
| Cinnamic acid | Good | 148.16 | Optimal | Soluble | Yes | No | High | 0.85 | 1.67 | Undefined | Non-mutagenic | Non-inhibitor |
| Picroside I | Moderate | 492.47 | Optimal | Soluble | No | Yes | Low | 0.55 | 6.12 | Undefined | Undefined | Non-inhibitor |
| Cinnamaldehyde | Good | 132.16 | Optimal | Soluble | Yes | No | High | 0.55 | 1.65 | Undefined | Undefined | Undefined |
| Galangin | Good | 270.24 | Optimal | Insoluble | No | No | High | 0.55 | 3.12 | Undefined | Mutagenic | Non-inhibitor |
| Baicalin | Bad | 446.36 | Optimal | Soluble | No | Yes | Low | 0.11 | 5.09 | Undefined | Undefined | Undefined |
| Chrysin | Good | 254.24 | Optimal | Insoluble | Yes | No | High | 0.55 | 2.93 | Undefined | Undefined | Non-inhibitor |
| Baicalein | Good | 270.24 | Optimal | Soluble | No | No | High | 0.55 | 3.02 | Undefined | Mutagenic | Non-inhibitor |
| Dicumarol | Good | 336.29 | Optimal | Soluble | No | No | High | 0.55 | 3.37 | Undefined | Undefined | Non-inhibitor |
| Indirubin | Good | 262.26 | Optimal | Insoluble | Yes | No | High | 0.55 | 2.84 | Undefined | Undefined | Non-inhibitor |
| Alizarin | Good | 240.21 | Optimal | Insoluble | Yes | No | High | 0.55 | 2.35 | Undefined | Mutagenic | Non-inhibitor |
| Rhein | Good | 284.22 | Optimal | Soluble | No | No | High | 0.56 | 2.58 | Undefined | Mutagenic | Non-inhibitor |
| Aloeemodin | Good | 270.24 | Optimal | Soluble | No | No | High | 0.55 | 2.6 | Undefined | Mutagenic | Non-inhibitor |
| Barbaloin | Moderate | 418.39 | Optimal | Soluble | No | No | Low | 0.55 | 4.97 | Stable in HLM | Undefined | Non-inhibitor |
| Sennoside A | Bad | 862.74 | Optimal | Soluble | No | Yes | Low | 0.11 | 7.51 | Undefined | Undefined | Non-inhibitor |
| Sennoside B | Bad | 862.74 | Optimal | Soluble | No | Yes | Low | 0.11 | 7.51 | Undefined | Undefined | Non-inhibitor |
| Juglone | Good | 174.15 | Optimal | Soluble | Yes | No | High | 0.55 | 2.31 | Undefined | Mutagenic | Undefined |
| Angelicin | Good | 186.16 | Optimal | Soluble | Yes | No | High | 0.55 | 3.07 | Undefined | Undefined | Non-inhibitor |
| Catalpol | Bad | 362.33 | Very hydrophilic | Soluble | No | Yes | Low | 0.55 | 5.72 | Undefined | Undefined | Non-inhibitor |
| Adenosine | Good | 267.24 | Hydrophilic | Soluble | No | No | Low | 0.55 | 3.86 | Undefined | Undefined | Non-inhibitor |
| Chlorogenic acid | Moderate | 354.31 | Optimal | Soluble | No | No | Low | 0.11 | 4.16 | Undefined | Non-mutagenic | Non-inhibitor |
| Cianidanol | Good | 290.27 | Optimal | Soluble | No | Yes | High | 0.55 | 3.5 | Stable in HLM | Undefined | Non-inhibitor |
| Cordycepin | Good | 251.24 | Optimal | Soluble | No | No | High | 0.55 | 3.67 | Undefined | Undefined | Non-inhibitor |
| Liquiritin | Good | 418.39 | Optimal | Soluble | No | Yes | Low | 0.55 | 4.91 | Undefined | Undefined | Non-inhibitor |
| Naringetol | Good | 272.25 | Optimal | Soluble | No | Yes | High | 0.55 | 3.01 | Undefined | Non-mutagenic | Non-inhibitor |
| Brazilin | Good | 286.28 | Optimal | Soluble | No | Yes | High | 0.55 | 3.7 | Undefined | Undefined | Undefined |
| Trifolirhizin | Good | 446.4 | Optimal | Soluble | No | Yes | High | 0.55 | 5.29 | Undefined | Undefined | Non-inhibitor |
| Sesamin | Good | 354.35 | Optimal | Soluble | Yes | No | High | 0.55 | 4.12 | Undefined | Undefined | Undefined |
| Cytisine | Good | 190.24 | Optimal | Soluble | No | No | High | 0.55 | 3.54 | Undefined | Undefined | Undefined |
| Oxymatrine | Good | 264.36 | Optimal | Soluble | Yes | Yes | High | 0.55 | 3.5 | Undefined | Undefined | Undefined |
| Matrine | Good | 248.36 | Optimal | Soluble | Yes | No | High | 0.55 | 3.2 | Undefined | Undefined | Non-inhibitor |
| Sophocarpine | Good | 246.35 | Optimal | Soluble | Yes | No | High | 0.55 | 4.38 | Undefined | Undefined | Non-inhibitor |
| Piperine | Good | 285.34 | Optimal | Insoluble | Yes | No | High | 0.55 | 2.92 | Undefined | Undefined | Undefined |
| Securinine | Good | 217.26 | Optimal | Soluble | Yes | No | High | 0.55 | 4.83 | Undefined | Undefined | Undefined |
| Anabasine | Good | 162.23 | Optimal | Soluble | Yes | No | High | 0.55 | 2.07 | Undefined | Undefined | Non-inhibitor |
| Rutaecarpine | Good | 287.32 | Optimal | Highly insoluble | Yes | Yes | High | 0.55 | 2.78 | Undefined | Undefined | Undefined |
| Lycorine | Good | 287.31 | Optimal | Soluble | No | Yes | High | 0.55 | 4.2 | Undefined | Undefined | Undefined |
| Strychnine | Good | 334.41 | Optimal | Soluble | Yes | No | High | 0.55 | 5.1 | Undefined | Non-mutagenic | Undefined |
| Higenamine | Good | 271.31 | Optimal | Soluble | No | Yes | High | 0.55 | 2.62 | Undefined | Undefined | Undefined |
| Tutin | Good | 294.3 | Optimal | Soluble | No | Yes | High | 0.55 | 5.74 | Undefined | Mutagenic | Undefined |
| Coriamyrtin | Good | 278.3 | Optimal | Soluble | No | Yes | High | 0.55 | 5.61 | Undefined | Undefined | Undefined |
| beta-Elemene | Moderate | 204.35 | Very lipophilic | Highly insoluble | No | No | Low | 0.55 | 3.63 | Undefined | Non-mutagenic | Undefined |
| Betulonic acid | Moderate | 454.68 | Very lipophilic | Highly insoluble | No | No | Low | 0.85 | 5.49 | Undefined | Non-mutagenic | Non-inhibitor |
| Betulinic acid | Moderate | 456.7 | Very lipophilic | Highly insoluble | No | No | Low | 0.85 | 5.63 | Undefined | Non-mutagenic | Non-inhibitor |
| Betulin | Moderate | 442.72 | Very lipophilic | Highly insoluble | No | No | Low | 0.55 | 5.68 | Undefined | Undefined | Undefined |
| Crocetin | Good | 328.4 | Optimal | Soluble | No | No | High | 0.85 | 3.99 | Unstable in HLM | Non-mutagenic | Undefined |
| Crocin II | Bad | 814.82 | Optimal | Insoluble | No | Yes | Low | 0.17 | 8.44 | Undefined | Undefined | Undefined |
| alpha-Crocin | Bad | 976.96 | Very hydrophilic | Insoluble | No | Yes | Low | 0.17 | 9.54 | Undefined | Undefined | Non-inhibitor |
| Pseudolaric acid B | Good | 432.46 | Optimal | Soluble | No | No | High | 0.56 | 5.82 | Undefined | Undefined | Undefined |
| Alkannin | Good | 288.3 | Optimal | Soluble | No | No | High | 0.55 | 3.55 | Undefined | Mutagenic | Non-inhibitor |
| Acetylshikonin | Good | 330.33 | Lipophilic | Insoluble | No | No | High | 0.55 | 3.85 | Undefined | Undefined | Undefined |
| beta,beta-Dimethylacrylshikonin | Moderate | 370.4 | Very lipophilic | Highly insoluble | No | No | High | 0.55 | 4.13 | Undefined | Undefined | Undefined |
| Isobavachalcone | Good | 324.37 | Lipophilic | Highly insoluble | No | No | High | 0.55 | 3.03 | Undefined | Undefined | Undefined |
| Osthole | Good | 244.29 | Optimal | Highly insoluble | Yes | No | High | 0.55 | 3.13 | Undefined | Undefined | Undefined |
| Lapachol | Good | 242.27 | Optimal | Soluble | Yes | No | High | 0.85 | 2.98 | Undefined | Undefined | Non-inhibitor |
| Citral | Good | 152.23 | Optimal | Soluble | Yes | No | High | 0.55 | 2.49 | Undefined | Non-mutagenic | Undefined |
| Geraniol | Good | 154.25 | Optimal | Soluble | Yes | No | High | 0.55 | 2.58 | Undefined | Non-mutagenic | Undefined |
| Bakuchiol | Moderate | 256.38 | Very lipophilic | Insoluble | Yes | No | High | 0.55 | 3.13 | Undefined | Non-mutagenic | Undefined |
| Ginsenoside Rg1 | Bad | 801.01 | Optimal | Insoluble | No | Yes | Low | 0.17 | 9.21 | Undefined | Non-mutagenic | Non-inhibitor |
| Ginsenoside Rf | Bad | 801.01 | Optimal | Insoluble | No | Yes | Low | 0.17 | 9.25 | Undefined | Non-mutagenic | Non-inhibitor |
| Notoginsenoside R1 | Bad | 933.13 | Optimal | Insoluble | No | Yes | Low | 0.17 | 10 | Undefined | Non-mutagenic | Non-inhibitor |
| Ginsenoside Rb1 | Bad | 1109.29 | Optimal | Highly insoluble | No | Yes | Low | 0.17 | 10 | Undefined | Non-mutagenic | Non-inhibitor |
| Ginsenoside Rb2 | Bad | 1079.27 | Optimal | Insoluble | No | Yes | Low | 0.17 | 10 | Undefined | Non-mutagenic | Non-inhibitor |
| Ginsenoside Rc | Bad | 1079.27 | Optimal | Highly insoluble | No | Yes | Low | 0.17 | 10 | Undefined | Non-mutagenic | Non-inhibitor |
| Gambogic acid | Bad | 628.75 | Very lipophilic | Highly insoluble | No | Yes | Low | 0.56 | 7.66 | Undefined | Undefined | Undefined |
| Imperatorin | Good | 270.28 | Optimal | Insoluble | Yes | No | High | 0.55 | 3.22 | Undefined | Mutagenic | Undefined |
| Isoimperatorin | Good | 270.28 | Optimal | Insoluble | Yes | No | High | 0.55 | 3.24 | Undefined | Undefined | Undefined |
| Paeonol | Good | 166.17 | Optimal | Soluble | Yes | No | High | 0.55 | 1.28 | Undefined | Undefined | Non-inhibitor |
| Colchicine | Good | 399.44 | Optimal | Soluble | No | Yes | High | 0.55 | 3.87 | Undefined | Non-mutagenic | Undefined |
| Cucurbitacin E | Moderate | 556.69 | Optimal | Highly insoluble | No | Yes | Low | 0.55 | 6.74 | Undefined | Undefined | Non-inhibitor |
| Cucurbitacin B | Moderate | 558.7 | Optimal | Highly insoluble | No | Yes | Low | 0.55 | 6.79 | Undefined | Undefined | Non-inhibitor |
| Cinobufagin | Good | 442.54 | Optimal | Highly insoluble | No | Yes | High | 0.55 | 6.48 | Undefined | Undefined | Undefined |
| Toosendanin | Bad | 574.62 | Optimal | Soluble | No | Yes | Low | 0.17 | 7.27 | Undefined | Undefined | Undefined |
| (-)-Bornyl acetate | Good | 196.29 | Optimal | Soluble | Yes | No | High | 0.55 | 3.64 | Undefined | Non-mutagenic | Non-inhibitor |
| Ephedrine | Good | 165.23 | Optimal | Soluble | Yes | No | High | 0.55 | 1.76 | Undefined | Non-mutagenic | Undefined |
| Ephedrine hydrochloride | Good | 201.69 | Optimal | Soluble | Yes | No | High | 0.55 | 1.86 | Undefined | Non-mutagenic | Undefined |
| Bilobalide | Good | 326.3 | Optimal | Soluble | No | Yes | High | 0.55 | 5.41 | Undefined | Undefined | Undefined |
| Cimifugin | Good | 306.31 | Optimal | Soluble | No | Yes | High | 0.55 | 3.85 | Undefined | Undefined | Undefined |
| Prim-O-glucosylcimifugin | Moderate | 468.45 | Optimal | Soluble | No | No | Low | 0.55 | 5.7 | Undefined | Undefined | Non-inhibitor |
| Harringtonine | Moderate | 531.59 | Optimal | Soluble | No | No | High | 0.55 | 6.31 | Undefined | Undefined | Undefined |
| Homoharringtonine | Moderate | 545.62 | Optimal | Soluble | No | No | High | 0.55 | 6.43 | Undefined | Undefined | Undefined |
| Capsaicin | Good | 305.41 | Optimal | Soluble | Yes | No | High | 0.55 | 2.32 | Undefined | Non-mutagenic | Undefined |
| Nagilactone C | Good | 362.37 | Optimal | Soluble | No | Yes | High | 0.55 | 5.36 | Undefined | Undefined | Undefined |
| Tripdiolide | Good | 376.4 | Optimal | Highly insoluble | No | Yes | High | 0.55 | 6.21 | Undefined | Mutagenic | Undefined |
| Triptonide | Good | 358.39 | Optimal | Highly insoluble | No | No | High | 0.55 | 5.94 | Undefined | Undefined | Non-inhibitor |
| Triptolide | Good | 360.4 | Optimal | Highly insoluble | No | Yes | High | 0.55 | 6.02 | Undefined | Mutagenic | Undefined |
| Lupulone | Moderate | 414.58 | Very lipophilic | Insoluble | No | Yes | Low | 0.85 | 5 | Undefined | Non-mutagenic | Undefined |
| Humulone | Good | 362.46 | Optimal | Soluble | No | Yes | High | 0.56 | 5.12 | Unstable in HLM | Non-mutagenic | Undefined |
| Cyclovirobuxine D | Good | 402.66 | Lipophilic | Soluble | Yes | Yes | High | 0.55 | 5.66 | Undefined | Undefined | Non-inhibitor |
| Baccharin | Bad | 562.61 | Optimal | Insoluble | No | Yes | Low | 0.17 | 7.91 | Undefined | Mutagenic | Undefined |
| Alisol A 24-acetate | Bad | 532.75 | Very lipophilic | Highly insoluble | No | Yes | High | 0.55 | 6.63 | Undefined | Undefined | Non-inhibitor |
| Sodium taurocholate | Moderate | 537.68 | Optimal | Soluble | No | Yes | Low | 0.55 | 5.95 | Stable in HLM | Undefined | Non-inhibitor |
| Cholic acid | Good | 408.57 | Optimal | Soluble | No | Yes | High | 0.56 | 5.03 | Undefined | Undefined | Non-inhibitor |
| Deoxycholic acid | Good | 392.57 | Optimal | Soluble | No | Yes | High | 0.56 | 4.76 | Undefined | Undefined | Non-inhibitor |
| Chenodeoxycholic acid | Good | 392.57 | Optimal | Soluble | No | Yes | High | 0.56 | 4.93 | Undefined | Undefined | Non-inhibitor |
| Ergonovine | Good | 325.4 | Optimal | Soluble | No | Yes | High | 0.55 | 4.27 | Undefined | Mutagenic | Undefined |
| Aescine | Bad | 1131.26 | Optimal | Soluble | No | Yes | Low | 0.11 | 10 | Undefined | Non-mutagenic | Non-inhibitor |
| Praeruptorin A | Good | 386.4 | Optimal | Insoluble | No | No | High | 0.55 | 4.55 | Undefined | Undefined | Undefined |
| Fucosterol | Moderate | 412.69 | Very lipophilic | Highly insoluble | No | No | Low | 0.55 | 6.15 | Undefined | Non-mutagenic | Undefined |
| Oleuropein | Bad | 540.51 | Optimal | Soluble | No | No | Low | 0.11 | 6.22 | Undefined | Undefined | Undefined |
| Huperzine A | Good | 242.32 | Optimal | Soluble | Yes | Yes | High | 0.55 | 4.26 | Undefined | Undefined | Undefined |
| Lobetyolin | Moderate | 396.43 | Optimal | Soluble | No | Yes | Low | 0.55 | 6.15 | Undefined | Undefined | Non-inhibitor |
| alpha-Asarone | Good | 208.25 | Optimal | Soluble | Yes | No | High | 0.55 | 2.39 | Undefined | Undefined | Undefined |
| Anethole | Good | 148.2 | Optimal | Soluble | Yes | No | High | 0.55 | 1.47 | Undefined | Non-mutagenic | Non-inhibitor |
| Ergotamine | Moderate | 581.66 | Optimal | Soluble | No | Yes | High | 0.55 | 6.1 | Undefined | Non-mutagenic | Undefined |
| Astragaloside IV | Bad | 784.97 | Optimal | Highly insoluble | No | Yes | Low | 0.17 | 9.73 | Undefined | Non-mutagenic | Non-inhibitor |
| Oxypeucedanin | Good | 286.28 | Optimal | Insoluble | Yes | Yes | High | 0.55 | 3.73 | Undefined | Undefined | Undefined |
| Mollugin | Good | 284.31 | Lipophilic | Highly insoluble | Yes | No | High | 0.55 | 3.41 | Undefined | Mutagenic | Undefined |
| Cucurbitacin I | Moderate | 514.65 | Optimal | Insoluble | No | Yes | Low | 0.55 | 6.61 | Undefined | Undefined | Non-inhibitor |
| Cucurbitacin D | Moderate | 516.67 | Optimal | Insoluble | No | Yes | High | 0.55 | 6.65 | Undefined | Undefined | Non-inhibitor |
| Limonin | Good | 470.51 | Optimal | Insoluble | No | No | High | 0.55 | 6.49 | Undefined | Undefined | Undefined |
| Miroestrol | Good | 358.39 | Optimal | Soluble | No | Yes | High | 0.55 | 5.29 | Stable in HLM | Undefined | Non-inhibitor |
| Eucalyptol | Good | 154.25 | Optimal | Soluble | Yes | No | High | 0.55 | 3.65 | Undefined | Non-mutagenic | Non-inhibitor |
| D-Camphor | Good | 152.23 | Optimal | Soluble | Yes | No | High | 0.55 | 3.22 | Undefined | Non-mutagenic | Non-inhibitor |
| (-)-Isoborneol | Good | 154.25 | Optimal | Soluble | Yes | No | High | 0.55 | 3.43 | Undefined | Non-mutagenic | Non-inhibitor |
| 3-Bornanol | Good | 154.25 | Optimal | Soluble | Yes | No | High | 0.55 | 3.43 | Undefined | Non-mutagenic | Non-inhibitor |
| Glycyrrhetic acid | Moderate | 470.68 | Very lipophilic | Highly insoluble | No | Yes | High | 0.85 | 6.08 | Undefined | Non-mutagenic | Non-inhibitor |
| Glycyrrhizic acid | Bad | 822.93 | Optimal | Soluble | No | Yes | Low | 0.11 | 8.84 | Undefined | Non-mutagenic | Non-inhibitor |
| Harpagide | Bad | 364.35 | Very hydrophilic | Soluble | No | Yes | Low | 0.55 | 5.76 | Undefined | Undefined | Non-inhibitor |
| Harpagoside | Bad | 494.49 | Optimal | Soluble | No | No | Low | 0.17 | 6.13 | Undefined | Undefined | Non-inhibitor |
| Oridonin | Good | 364.43 | Optimal | Insoluble | No | Yes | High | 0.55 | 6.68 | Undefined | Undefined | Non-inhibitor |
| Ponicidin | Good | 362.42 | Optimal | Insoluble | No | Yes | High | 0.55 | 6.34 | Undefined | Undefined | Undefined |
| Polygalacic acid | Bad | 504.7 | Very lipophilic | Insoluble | No | Yes | High | 0.56 | 6.59 | Undefined | Non-mutagenic | Non-inhibitor |
| Oleanolic acid | Moderate | 456.7 | Very lipophilic | Highly insoluble | No | No | Low | 0.85 | 6.08 | Undefined | Non-mutagenic | Non-inhibitor |
| Hederagenin | Moderate | 472.7 | Very lipophilic | Highly insoluble | No | Yes | High | 0.56 | 6.24 | Undefined | Non-mutagenic | Non-inhibitor |
| Kirenol | Good | 338.48 | Optimal | Insoluble | No | Yes | High | 0.55 | 5.42 | Undefined | Undefined | Non-inhibitor |
| Psorospermin | Good | 340.33 | Optimal | Insoluble | No | No | High | 0.55 | 4.21 | Undefined | Mutagenic | Undefined |
| Farrerol | Good | 300.31 | Optimal | Insoluble | No | Yes | High | 0.55 | 3.26 | Undefined | Non-mutagenic | Non-inhibitor |
| Celastrol | Moderate | 450.61 | Very lipophilic | Highly insoluble | No | Yes | Low | 0.85 | 6.28 | Undefined | Non-mutagenic | Non-inhibitor |
| Pristimerin | Moderate | 464.64 | Very lipophilic | Highly insoluble | No | Yes | Low | 0.55 | 6.3 | Undefined | Non-mutagenic | Non-inhibitor |
| Maltol | Good | 126.11 | Optimal | Soluble | Yes | No | High | 0.55 | 2.39 | Undefined | Mutagenic | Non-inhibitor |
| Bruceantin | Bad | 548.58 | Optimal | Insoluble | No | Yes | Low | 0.17 | 6.77 | Undefined | Undefined | Undefined |
| Brusatol | Bad | 520.53 | Optimal | Insoluble | No | Yes | Low | 0.17 | 6.5 | Undefined | Undefined | Undefined |
| Withaferin A | Good | 470.6 | Optimal | Highly insoluble | No | Yes | High | 0.55 | 6.83 | Undefined | Undefined | Undefined |
| Carvacrol | Good | 150.22 | Optimal | Soluble | Yes | No | High | 0.55 | 1 | Undefined | Undefined | Non-inhibitor |
| Pyrolin | Good | 124.14 | Optimal | Soluble | Yes | No | High | 0.55 | 1 | Undefined | Non-mutagenic | Non-inhibitor |
| Dracorhodin | Good | 266.29 | Optimal | Highly insoluble | Yes | No | High | 0.55 | 3.4 | Undefined | Undefined | Non-inhibitor |
| Ligustrazine | Good | 136.19 | Optimal | Soluble | Yes | No | High | 0.55 | 1.83 | Stable in HLM | Non-mutagenic | Non-inhibitor |
| Bilirubin | Bad | 584.66 | Optimal | Soluble | No | Yes | Low | 0.11 | 5.23 | Unstable in HLM | Non-mutagenic | Undefined |
| Fraxinellone | Good | 232.28 | Optimal | Insoluble | Yes | No | High | 0.55 | 3.91 | Undefined | Undefined | Undefined |
| Paclitaxel | Bad | 853.91 | Optimal | Highly insoluble | No | Yes | Low | 0.17 | 8.34 | Undefined | Non-mutagenic | Undefined |
| Lactucin | Good | 276.28 | Optimal | Soluble | No | No | High | 0.55 | 4.49 | Undefined | Undefined | Non-inhibitor |
| Atractylenolide I | Good | 230.3 | Optimal | Insoluble | Yes | No | High | 0.55 | 4.27 | Undefined | Undefined | Undefined |
| Thymol | Good | 150.22 | Optimal | Soluble | Yes | No | High | 0.55 | 1 | Undefined | Undefined | Non-inhibitor |
| Bruceine D | Good | 410.42 | Optimal | Soluble | No | Yes | Low | 0.55 | 5.96 | Undefined | Undefined | Non-inhibitor |
| Plumbagin | Good | 188.18 | Optimal | Soluble | Yes | No | High | 0.55 | 2.41 | Undefined | Mutagenic | Non-inhibitor |
| Gossypol | Bad | 518.55 | Very lipophilic | Highly insoluble | No | No | Low | 0.17 | 3.55 | Undefined | Undefined | Non-inhibitor |
| Emodin | Good | 270.24 | Optimal | Insoluble | No | No | High | 0.55 | 2.57 | Undefined | Mutagenic | Non-inhibitor |
| Physcion | Good | 284.26 | Optimal | Insoluble | No | No | High | 0.55 | 2.69 | Undefined | Mutagenic | Non-inhibitor |
| Chrysophanol | Good | 254.24 | Optimal | Insoluble | Yes | No | High | 0.55 | 2.47 | Undefined | Mutagenic | Non-inhibitor |
| Eupatolide | Good | 248.32 | Optimal | Insoluble | Yes | No | High | 0.55 | 4.66 | Unstable in HLM | Non-mutagenic | Undefined |
| Deoxyelephantopin | Good | 344.36 | Optimal | Insoluble | No | No | High | 0.55 | 5.87 | Unstable in HLM | Undefined | Non-inhibitor |
| Germacrone | Good | 218.33 | Lipophilic | Highly insoluble | Yes | No | High | 0.55 | 3.9 | Unstable in HLM | Non-mutagenic | Undefined |
| alpha-Bisabolol | Good | 222.37 | Lipophilic | Insoluble | Yes | No | High | 0.55 | 3.95 | Undefined | Non-mutagenic | Undefined |
| Cnicin | Good | 378.42 | Optimal | Soluble | No | No | High | 0.55 | 5.47 | Undefined | Undefined | Undefined |
| Costunolide | Good | 232.32 | Optimal | Highly insoluble | Yes | No | High | 0.55 | 4.29 | Unstable in HLM | Non-mutagenic | Undefined |
| Lipiferolide | Good | 306.35 | Optimal | Insoluble | Yes | No | High | 0.55 | 5.06 | Unstable in HLM | Undefined | Undefined |
| alpha-Eudesmol | Good | 222.37 | Lipophilic | Highly insoluble | Yes | No | High | 0.55 | 4.08 | Undefined | Non-mutagenic | Undefined |
| Linderane | Good | 260.29 | Optimal | Soluble | Yes | Yes | High | 0.55 | 5.35 | Undefined | Undefined | Undefined |
| Tanshinone IIA | Good | 294.34 | Lipophilic | Highly insoluble | Yes | Yes | High | 0.55 | 3.66 | Undefined | Undefined | Non-inhibitor |
| Harmine | Good | 212.25 | Optimal | Soluble | Yes | No | High | 0.55 | 1.66 | Undefined | Mutagenic | Undefined |
| Harmalol | Good | 200.24 | Optimal | Soluble | Yes | Yes | High | 0.55 | 2.51 | Undefined | Undefined | Undefined |
| Harmaline | Good | 214.26 | Optimal | Insoluble | Yes | Yes | High | 0.55 | 2.58 | Undefined | Undefined | Undefined |
| Corynoline | Good | 367.4 | Optimal | Soluble | Yes | No | High | 0.55 | 4.03 | Undefined | Undefined | Undefined |
| Cantharidin | Good | 196.2 | Optimal | Soluble | Yes | No | High | 0.55 | 3.48 | Undefined | Undefined | Non-inhibitor |
| Paeoniflorin | Moderate | 480.46 | Optimal | Soluble | No | Yes | Low | 0.55 | 5.51 | Undefined | Undefined | Non-inhibitor |
| Andrographolide | Good | 350.45 | Optimal | Soluble | No | Yes | High | 0.55 | 5.06 | Undefined | Undefined | Non-inhibitor |
| Resibufogenin | Good | 384.51 | Optimal | Highly insoluble | Yes | Yes | High | 0.55 | 5.98 | Undefined | Undefined | Undefined |
| Strophanthidin | Good | 404.5 | Optimal | Soluble | No | Yes | High | 0.55 | 5.42 | Undefined | Undefined | Undefined |
| Estriol | Good | 288.38 | Optimal | Insoluble | Yes | Yes | High | 0.55 | 3.74 | Stable in HLM | Non-mutagenic | Non-inhibitor |
| Estradiol | Good | 272.38 | Optimal | Highly insoluble | Yes | Yes | High | 0.55 | 3.49 | Stable in HLM | Non-mutagenic | Non-inhibitor |
| Isoalantolactone | Good | 232.32 | Optimal | Insoluble | Yes | No | High | 0.55 | 3.74 | Undefined | Undefined | Undefined |
| Friedelin | Moderate | 426.72 | Very lipophilic | Highly insoluble | No | No | Low | 0.55 | 5.17 | Undefined | Non-mutagenic | Non-inhibitor |
| Ginkgolide A | Good | 408.4 | Optimal | Soluble | No | Yes | High | 0.55 | 6.28 | Undefined | Undefined | Non-inhibitor |
| Ginkgolide B | Good | 424.4 | Optimal | Soluble | No | Yes | Low | 0.55 | 6.38 | Undefined | Undefined | Undefined |
| Ginkgolide C | Moderate | 440.4 | Optimal | Soluble | No | Yes | Low | 0.55 | 6.48 | Undefined | Undefined | Undefined |
| Monocrotaline | Good | 325.36 | Optimal | Soluble | No | Yes | High | 0.55 | 5.26 | Undefined | Non-mutagenic | Undefined |
| Icariin | Bad | 676.66 | Optimal | Insoluble | No | Yes | Low | 0.17 | 7.24 | Undefined | Mutagenic | Non-inhibitor |
| Quercitrin | Bad | 448.38 | Optimal | Soluble | No | No | Low | 0.17 | 5.28 | Undefined | Mutagenic | Non-inhibitor |
| Kaempferitrin | Bad | 578.52 | Optimal | Soluble | No | Yes | Low | 0.17 | 6.48 | Undefined | Mutagenic | Non-inhibitor |
| Neohesperidin | Bad | 610.56 | Optimal | Soluble | No | Yes | Low | 0.17 | 6.36 | Undefined | Non-mutagenic | Undefined |
| Naringin | Bad | 580.53 | Optimal | Soluble | No | Yes | Low | 0.17 | 6.16 | Undefined | Undefined | Undefined |
| Raddeanin A | Bad | 897.1 | Very lipophilic | Insoluble | No | Yes | Low | 0.11 | 9.77 | Undefined | Non-mutagenic | Non-inhibitor |
| Ginsenoside Re | Bad | 947.15 | Optimal | Insoluble | No | Yes | Low | 0.17 | 10 | Undefined | Non-mutagenic | Non-inhibitor |
| Tubeimoside I | Bad | 1319.43 | Lipophilic | Insoluble | No | Yes | Low | 0.17 | 10 | Undefined | Non-mutagenic | Non-inhibitor |
| Platycodin D | Bad | 1225.32 | Hydrophilic | Soluble | No | Yes | Low | 0.17 | 10 | Undefined | Non-mutagenic | Non-inhibitor |
| Jujuboside B | Bad | 1045.21 | Lipophilic | Highly insoluble | No | Yes | Low | 0.17 | 10 | Undefined | Non-mutagenic | Non-inhibitor |
| Jujuboside A | Bad | 1207.35 | Optimal | Insoluble | No | Yes | Low | 0.17 | 10 | Undefined | Non-mutagenic | Non-inhibitor |
| Verbascoside | Bad | 624.59 | Optimal | Soluble | No | Yes | Low | 0.17 | 6.37 | Undefined | Undefined | Non-inhibitor |
| Echinacoside | Bad | 786.73 | Hydrophilic | Soluble | No | No | Low | 0.17 | 7.49 | Undefined | Undefined | Non-inhibitor |
| Astilbin | Bad | 450.39 | Optimal | Soluble | No | Yes | Low | 0.17 | 5.27 | Undefined | Undefined | Non-inhibitor |
| Buddlejasaponin IV | Bad | 943.12 | Optimal | Insoluble | No | Yes | Low | 0.17 | 10 | Undefined | Non-mutagenic | Non-inhibitor |
| Pectolinarin | Bad | 622.57 | Optimal | Soluble | No | Yes | Low | 0.17 | 6.63 | Undefined | Undefined | Undefined |
| Rutin | Bad | 610.52 | Optimal | Soluble | No | Yes | Low | 0.17 | 6.52 | Undefined | Mutagenic | Non-inhibitor |
| Typhaneoside | Bad | 770.69 | Optimal | Soluble | No | Yes | Low | 0.17 | 7.72 | Undefined | Mutagenic | Undefined |
| Robinin | Bad | 740.66 | Hydrophilic | Soluble | No | Yes | Low | 0.17 | 7.6 | Undefined | Mutagenic | Non-inhibitor |
| Linarin | Bad | 592.55 | Optimal | Soluble | No | Yes | Low | 0.17 | 6.43 | Undefined | Undefined | Non-inhibitor |
| Hesperidin | Bad | 610.56 | Optimal | Soluble | No | Yes | Low | 0.17 | 6.34 | Stable in HLM | Undefined | Undefined |
| Digoxin | Bad | 780.94 | Optimal | Insoluble | No | Yes | Low | 0.17 | 8.81 | Undefined | Non-mutagenic | Non-inhibitor |
| Divaricoside | Moderate | 534.68 | Optimal | Insoluble | No | Yes | High | 0.55 | 6.96 | Undefined | Undefined | Undefined |
| Loganin | Moderate | 390.38 | Very hydrophilic | Soluble | No | Yes | Low | 0.11 | 5.77 | Undefined | Undefined | Non-inhibitor |
| Friedelanol | Moderate | 428.73 | Very lipophilic | Highly insoluble | No | No | Low | 0.55 | 5.27 | Undefined | Non-mutagenic | Undefined |
| Alstonine | Good | 349.4 | Hydrophilic | Highly insoluble | Yes | Yes | High | 0.85 | 4.36 | Undefined | Undefined | Undefined |
| Maytansine | Bad | 692.2 | Optimal | Insoluble | No | Yes | Low | 0.17 | 8.45 | Undefined | Undefined | Undefined |
| Conessine | Moderate | 356.59 | Very lipophilic | Soluble | Yes | No | High | 0.55 | 5.84 | Undefined | Undefined | Undefined |
| Calotropin | Moderate | 532.62 | Optimal | Insoluble | No | Yes | High | 0.55 | 6.78 | Undefined | Undefined | Undefined |
| Schisandrin | Good | 432.51 | Optimal | Insoluble | Yes | No | High | 0.55 | 4.55 | Undefined | Non-mutagenic | Undefined |
| Schizandrin A | Good | 416.51 | Lipophilic | Insoluble | Yes | No | High | 0.55 | 4.51 | Unstable in HLM | Non-mutagenic | Inhibitor |
| Schisantherin A | Moderate | 536.57 | Lipophilic | Insoluble | No | Yes | High | 0.55 | 5.33 | Undefined | Undefined | Undefined |
| Schisandrin B | Good | 400.46 | Lipophilic | Insoluble | Yes | No | High | 0.55 | 4.56 | Undefined | Undefined | Inhibitor |
| Jatrophon | Good | 312.4 | Optimal | Insoluble | Yes | No | High | 0.85 | 6.48 | Undefined | Undefined | Undefined |
| Menthol | Good | 156.27 | Optimal | Soluble | Yes | No | High | 0.55 | 2.63 | Undefined | Non-mutagenic | Non-inhibitor |
| Curdione | Good | 236.35 | Optimal | Soluble | Yes | No | High | 0.55 | 4.44 | Undefined | Non-mutagenic | Undefined |
| Polyphyllin VI | Bad | 738.9 | Lipophilic | Insoluble | No | Yes | Low | 0.17 | 9.3 | Undefined | Non-mutagenic | Non-inhibitor |
| Solasodine | Moderate | 413.64 | Very lipophilic | Soluble | Yes | Yes | High | 0.55 | 6.56 | Undefined | Non-mutagenic | Undefined |
| Diosgenin | Moderate | 414.62 | Very lipophilic | Highly insoluble | Yes | No | High | 0.55 | 6.94 | Undefined | Non-mutagenic | Undefined |
| Dioscin | Bad | 869.04 | Very lipophilic | Insoluble | No | Yes | Low | 0.17 | 10 | Undefined | Non-mutagenic | Non-inhibitor |
| Polyphyllin I | Bad | 855.02 | Very lipophilic | Insoluble | No | Yes | Low | 0.17 | 10 | Undefined | Non-mutagenic | Non-inhibitor |
| Polyphyllin II | Bad | 855.02 | Very lipophilic | Insoluble | No | Yes | Low | 0.17 | 10 | Undefined | Non-mutagenic | Non-inhibitor |
| Sarsasapogenin | Moderate | 416.64 | Very lipophilic | Highly insoluble | Yes | No | High | 0.55 | 6.88 | Stable in HLM | Non-mutagenic | Undefined |
| Tomatine | Bad | 1034.19 | Optimal | Soluble | No | Yes | Low | 0.17 | 10 | Undefined | Non-mutagenic | Non-inhibitor |
| Asiaticoside | Bad | 959.12 | Optimal | Highly insoluble | No | Yes | Low | 0.17 | 10 | Undefined | Non-mutagenic | Non-inhibitor |
| Ursolic acid | Moderate | 456.7 | Very lipophilic | Highly insoluble | No | No | Low | 0.85 | 6.21 | Undefined | Non-mutagenic | Non-inhibitor |
| Artemisinin | Good | 282.33 | Optimal | Insoluble | Yes | No | High | 0.55 | 6.13 | Undefined | Undefined | Undefined |
| Ambrosin | Good | 246.3 | Optimal | Soluble | Yes | No | High | 0.55 | 4.34 | Undefined | Non-mutagenic | Non-inhibitor |
| Cevane-3,6,14,16,20-pentol | Good | 463.65 | Optimal | Soluble | No | Yes | High | 0.55 | 5.65 | Undefined | Undefined | Non-inhibitor |
| alpha-Solanin | Bad | 868.06 | Lipophilic | Soluble | No | Yes | Low | 0.17 | 9.47 | Undefined | Non-mutagenic | Non-inhibitor |
| Imperialine | Good | 429.64 | Optimal | Soluble | Yes | Yes | High | 0.55 | 5.17 | Undefined | Undefined | Undefined |
| Peimine | Good | 431.65 | Lipophilic | Soluble | Yes | Yes | High | 0.55 | 5.35 | Undefined | Undefined | Undefined |
| Hupehenine | Moderate | 415.65 | Very lipophilic | Soluble | Yes | Yes | High | 0.55 | 5.09 | Undefined | Undefined | Undefined |
| Curcumenol | Good | 234.33 | Optimal | Soluble | Yes | No | High | 0.55 | 5.73 | Undefined | Undefined | Undefined |
| Curcumol | Good | 236.35 | Optimal | Soluble | Yes | No | High | 0.55 | 5.52 | Undefined | Undefined | Undefined |
| Alantolactone | Good | 232.32 | Optimal | Insoluble | Yes | No | High | 0.55 | 4.19 | Undefined | Undefined | Undefined |
| Gnidimacrin | Bad | 774.89 | Very lipophilic | Insoluble | No | Yes | Low | 0.17 | 9.05 | Undefined | Undefined | Undefined |
| Muscone | Moderate | 238.41 | Very lipophilic | Insoluble | Yes | No | High | 0.55 | 3.09 | Undefined | Non-mutagenic | Undefined |
| Simalikilactone D | Good | 478.53 | Optimal | Soluble | No | Yes | High | 0.55 | 6.53 | Undefined | Undefined | Undefined |
| Trilobolide | Moderate | 522.58 | Optimal | Insoluble | No | No | Low | 0.55 | 6.38 | Unstable in HLM | Undefined | Undefined |
| Hirudin | Bad | 1468.52 | Hydrophilic | Soluble | No | Yes | Low | 0.11 | 10 | Undefined | Non-mutagenic | Non-inhibitor |
| beta-Sitosterol | Moderate | 414.71 | Very lipophilic | Highly insoluble | No | No | Low | 0.55 | 6.3 | Unstable in HLM | Non-mutagenic | Undefined |
| 10-Hydroxycamptothecin | Good | 364.35 | Optimal | Soluble | No | Yes | High | 0.55 | 3.84 | Undefined | Undefined | Undefined |
| Camptothecine | Good | 348.35 | Optimal | Insoluble | No | Yes | High | 0.55 | 3.84 | Undefined | Undefined | Undefined |
| Vincaleukoblastine | Bad | 810.97 | Optimal | Soluble | No | Yes | Low | 0.17 | 9.65 | Undefined | Non-mutagenic | Undefined |
| Vincristine | Bad | 824.96 | Optimal | Soluble | No | Yes | Low | 0.17 | 9.59 | Undefined | Non-mutagenic | Undefined |
| Vincamine | Good | 354.44 | Optimal | Soluble | Yes | Yes | High | 0.55 | 4.49 | Undefined | Undefined | Undefined |
| Voacanginine | Bad | 704.9 | Very lipophilic | Soluble | No | No | High | 0.55 | 7.99 | Undefined | Undefined | Undefined |
| Tabernaemontanin | Good | 354.44 | Optimal | Soluble | Yes | Yes | High | 0.55 | 4.71 | Undefined | Undefined | Undefined |
| Rhynchophylline | Good | 384.47 | Optimal | Soluble | Yes | No | High | 0.85 | 4.78 | Undefined | Undefined | Undefined |
| Emetine | Good | 480.64 | Lipophilic | Soluble | Yes | Yes | High | 0.55 | 4.87 | Undefined | Non-mutagenic | Inhibitor |
| Agrimophol | Good | 474.54 | Lipophilic | Soluble | No | Yes | Low | 0.56 | 5.1 | Undefined | Undefined | Non-inhibitor |
| 6-Gingerol | Good | 294.39 | Optimal | Soluble | Yes | No | High | 0.55 | 2.81 | Undefined | Non-mutagenic | Undefined |
| Trilinolein | Bad | 879.38 | Very lipophilic | Highly insoluble | No | Yes | Low | 0.17 | 8.46 | Unstable in HLM | Non-mutagenic | Undefined |
| Ginkgolic acid | Moderate | 346.5 | Very lipophilic | Soluble | No | No | High | 0.85 | 3.12 | Undefined | Non-mutagenic | Non-inhibitor |
| Coixenolide | Bad | 590.96 | Very lipophilic | Highly insoluble | No | Yes | Low | 0.17 | 6.43 | Unstable in HLM | Non-mutagenic | Inhibitor |
| 2-Undecanone | Good | 170.29 | Optimal | Insoluble | Yes | No | High | 0.55 | 1.72 | Undefined | Non-mutagenic | Non-inhibitor |
| Houttuynin | Good | 198.3 | Optimal | Soluble | Yes | No | High | 0.55 | 1.76 | Undefined | Non-mutagenic | Undefined |
| Embelin | Moderate | 294.39 | Very lipophilic | Soluble | Yes | No | High | 0.85 | 3.66 | Undefined | Undefined | Non-inhibitor |
| Ginkgoneolic acid | Moderate | 320.47 | Very lipophilic | Highly insoluble | No | No | High | 0.85 | 2.74 | Undefined | Non-mutagenic | Undefined |
| Aconitine | Bad | 645.74 | Optimal | Soluble | No | Yes | Low | 0.17 | 7.43 | Undefined | Undefined | Non-inhibitor |
| Songorine | Good | 357.49 | Optimal | Soluble | Yes | Yes | High | 0.55 | 5.96 | Undefined | Undefined | Non-inhibitor |
| Arecoline | Good | 155.19 | Optimal | Soluble | No | No | High | 0.55 | 2.64 | Undefined | Undefined | Non-inhibitor |
| Gramine | Good | 174.24 | Optimal | Soluble | Yes | No | High | 0.55 | 1.44 | Undefined | Non-mutagenic | Undefined |
| Thaspine | Good | 369.37 | Optimal | Soluble | No | No | High | 0.55 | 3.42 | Undefined | Undefined | Undefined |
| Thaspine hydrochloride | Good | 405.83 | Optimal | Soluble | No | Yes | High | 0.55 | 3.49 | Undefined | Undefined | Undefined |
| Lobeline | Good | 337.46 | Optimal | Soluble | Yes | No | High | 0.55 | 3.23 | Undefined | Non-mutagenic | Undefined |
| Caffeine | Good | 194.19 | Optimal | Soluble | No | No | High | 0.55 | 2.03 | Undefined | Non-mutagenic | Non-inhibitor |
| Theophylline | Good | 180.16 | Optimal | Soluble | No | No | High | 0.55 | 1.87 | Undefined | Non-mutagenic | Non-inhibitor |
| Evodiamine | Good | 303.36 | Optimal | Highly insoluble | Yes | Yes | High | 0.55 | 3.19 | Undefined | Undefined | Undefined |
| Anisodamine | Good | 305.37 | Optimal | Soluble | No | No | High | 0.55 | 4.65 | Stable in HLM | Non-mutagenic | Non-inhibitor |
| Anisodine | Good | 319.35 | Optimal | Soluble | No | No | High | 0.55 | 4.11 | Stable in HLM | Undefined | Non-inhibitor |
| Scopolamine | Good | 303.35 | Optimal | Soluble | No | No | High | 0.55 | 4.03 | Stable in HLM | Non-mutagenic | Undefined |
| Scopolamine hydrobromide | Good | 384.26 | Optimal | Soluble | Yes | Yes | High | 0.55 | 4.13 | Stable in HLM | Non-mutagenic | Undefined |
| Atropine | Good | 289.37 | Optimal | Soluble | Yes | No | High | 0.55 | 4.33 | Stable in HLM | Non-mutagenic | Undefined |
| Atropine sulphate | Good | 676.82 | Optimal | Soluble | No | No | Low | 0.17 | 7.17 | Stable in HLM | Non-mutagenic | Undefined |
| Gelsemine | Good | 322.4 | Optimal | Soluble | Yes | Yes | High | 0.55 | 4.78 | Undefined | Undefined | Undefined |
| Chelidonine | Good | 353.37 | Optimal | Soluble | Yes | No | High | 0.55 | 3.92 | Undefined | Undefined | Undefined |
| Arecoline | Good | 155.19 | Optimal | Soluble | No | No | High | 0.55 | 2.65 | Undefined | Undefined | Non-inhibitor |
| Liensinine | Moderate | 610.74 | Lipophilic | Soluble | No | No | High | 0.55 | 5.09 | Undefined | Undefined | Inhibitor |
| Neferine | Bad | 624.77 | Very lipophilic | Soluble | No | No | High | 0.55 | 5.22 | Undefined | Undefined | Inhibitor |
| Dauricine | Bad | 624.77 | Very lipophilic | Soluble | No | No | High | 0.55 | 5.18 | Undefined | Undefined | Inhibitor |
| Pronuciferine | Good | 311.37 | Optimal | Soluble | Yes | No | High | 0.55 | 4.28 | Undefined | Undefined | Undefined |
| Fangchinoline | Moderate | 608.72 | Lipophilic | Insoluble | No | No | High | 0.55 | 6.89 | Undefined | Undefined | Inhibitor |
| Berbamine | Moderate | 608.72 | Lipophilic | Soluble | No | No | High | 0.55 | 6.89 | Undefined | Undefined | Inhibitor |
| Isotetrandrine | Moderate | 622.75 | Lipophilic | Insoluble | No | No | High | 0.55 | 7.01 | Undefined | Undefined | Inhibitor |
| Tubocurarine | Moderate | 609.73 | Optimal | Soluble | No | No | High | 0.55 | 6.92 | Undefined | Undefined | Inhibitor |
| Curine | Moderate | 594.7 | Lipophilic | Soluble | No | No | High | 0.55 | 6.76 | Undefined | Undefined | Inhibitor |
| Protopine | Good | 353.37 | Optimal | Soluble | Yes | Yes | High | 0.55 | 3.48 | Undefined | Undefined | Undefined |
| Stephanine | Good | 309.36 | Optimal | Soluble | Yes | Yes | High | 0.55 | 3.7 | Undefined | Mutagenic | Undefined |
| Cepharanthine | Bad | 606.71 | Very lipophilic | Soluble | No | No | High | 0.55 | 7.01 | Undefined | Mutagenic | Inhibitor |
| Galantamine | Good | 287.35 | Optimal | Soluble | Yes | Yes | High | 0.55 | 4.57 | Undefined | Undefined | Undefined |
| Morphine | Good | 285.34 | Optimal | Soluble | Yes | Yes | High | 0.55 | 4.78 | Stable in HLM | Non-mutagenic | Non-inhibitor |
| Codeine phosphate | Good | 397.36 | Optimal | Soluble | No | No | High | 0.55 | 5.24 | Stable in HLM | Non-mutagenic | Non-inhibitor |
| Sinomenine | Good | 329.39 | Optimal | Soluble | Yes | Yes | High | 0.55 | 4.52 | Undefined | Non-mutagenic | Undefined |
| Abrine | Good | 218.25 | Optimal | Soluble | Yes | No | High | 0.55 | 2.2 | Undefined | Non-mutagenic | Undefined |
| Colchamine | Good | 371.43 | Optimal | Soluble | Yes | Yes | High | 0.55 | 3.88 | Undefined | Non-mutagenic | Undefined |
| Synephrine | Good | 167.21 | Optimal | Soluble | No | No | High | 0.55 | 1.48 | Stable in HLM | Non-mutagenic | Non-inhibitor |
| Methyl salicylate | Good | 152.15 | Optimal | Soluble | Yes | No | High | 0.55 | 1.11 | Undefined | Non-mutagenic | Non-inhibitor |
| Genipin | Good | 226.23 | Optimal | Soluble | No | No | High | 0.56 | 4.48 | Undefined | Undefined | Non-inhibitor |
| Geniposide | Good | 388.37 | Hydrophilic | Soluble | No | No | Low | 0.11 | 5.8 | Undefined | Undefined | Non-inhibitor |
| Jacaranone | Good | 182.17 | Optimal | Soluble | No | No | High | 0.55 | 3.32 | Undefined | Undefined | Undefined |
| Fraxetin | Good | 208.17 | Optimal | Soluble | No | No | High | 0.55 | 2.87 | Undefined | Undefined | Undefined |
| Isofraxidin | Good | 222.19 | Optimal | Soluble | Yes | No | High | 0.55 | 3.02 | Undefined | Undefined | Undefined |
| Curculigoside | Moderate | 466.44 | Optimal | Soluble | No | Yes | Low | 0.55 | 5.05 | Stable in HLM | Undefined | Undefined |
| Isoferulic acid | Good | 194.18 | Optimal | Soluble | Yes | No | High | 0.85 | 1.9 | Undefined | Non-mutagenic | Undefined |
| Rhapontin | Moderate | 420.41 | Optimal | Soluble | No | Yes | Low | 0.55 | 4.97 | Undefined | Undefined | Non-inhibitor |
| Vitexicarpin | Good | 374.34 | Optimal | Insoluble | No | No | High | 0.55 | 3.71 | Undefined | Undefined | Undefined |
| Eupatorin | Good | 344.32 | Optimal | Insoluble | No | No | High | 0.55 | 3.43 | Undefined | Undefined | Undefined |
| Nobiletin | Good | 402.39 | Optimal | Insoluble | No | No | High | 0.55 | 3.9 | Unstable in HLM | Undefined | Undefined |
| Eupatilin | Good | 344.32 | Optimal | Insoluble | No | No | High | 0.55 | 3.39 | Undefined | Undefined | Non-inhibitor |
| Forsythoside D | Bad | 534.55 | Optimal | Soluble | No | Yes | Low | 0.17 | 5.94 | Undefined | Undefined | Undefined |
| Magnolin | Good | 416.46 | Optimal | Soluble | Yes | No | High | 0.55 | 4.44 | Undefined | Undefined | Undefined |
| Phillyrin | Bad | 534.55 | Optimal | Soluble | No | Yes | Low | 0.17 | 5.84 | Undefined | Undefined | Undefined |
| Methyleugenol | Good | 178.23 | Optimal | Soluble | Yes | No | High | 0.55 | 1.71 | Undefined | Undefined | Undefined |
| Papaverine | Good | 339.39 | Optimal | Insoluble | Yes | Yes | High | 0.55 | 2.62 | Unstable in HLM | Undefined | Inhibitor |
| Arctigenin | Good | 372.41 | Optimal | Insoluble | Yes | No | High | 0.55 | 3.43 | Undefined | Undefined | Undefined |
| Arctiin | Bad | 534.55 | Optimal | Soluble | No | Yes | Low | 0.17 | 5.59 | Undefined | Undefined | Undefined |
| Wogonin | Good | 284.26 | Optimal | Highly insoluble | No | No | High | 0.55 | 3.15 | Unstable in HLM | Undefined | Non-inhibitor |
| Daphnoretin | Good | 352.29 | Optimal | Insoluble | No | No | High | 0.55 | 3.48 | Undefined | Undefined | Undefined |
| Scopoletin | Good | 192.17 | Optimal | Soluble | Yes | No | High | 0.55 | 2.62 | Undefined | Undefined | Undefined |
| Scoparone | Good | 206.19 | Optimal | Soluble | Yes | No | High | 0.55 | 2.77 | Undefined | Undefined | Non-inhibitor |
| Scopolin | Good | 354.31 | Hydrophilic | Soluble | No | No | Low | 0.55 | 4.85 | Undefined | Undefined | Non-inhibitor |
| Tylophorine | Good | 393.48 | Lipophilic | Soluble | Yes | Yes | High | 0.55 | 3.48 | Undefined | Undefined | Undefined |
| Brucine | Good | 394.46 | Optimal | Soluble | Yes | No | High | 0.55 | 5.44 | Undefined | Non-mutagenic | Undefined |
| Tectoridin | Bad | 462.4 | Optimal | Soluble | No | Yes | Low | 0.17 | 5.3 | Undefined | Undefined | Non-inhibitor |
| Bergenin | Good | 328.27 | Optimal | Soluble | No | No | Low | 0.55 | 4.39 | Undefined | Undefined | Undefined |
| Vanillic acid | Good | 168.15 | Optimal | Soluble | No | No | High | 0.85 | 1.42 | Stable in HLM | Non-mutagenic | Non-inhibitor |
| Picroside II | Bad | 512.46 | Hydrophilic | Soluble | No | No | Low | 0.17 | 5.99 | Undefined | Undefined | Undefined |
| Curcumin | Good | 368.38 | Optimal | Insoluble | No | No | High | 0.55 | 2.97 | Undefined | Undefined | Undefined |
| Ferulic acid | Good | 194.18 | Optimal | Soluble | Yes | No | High | 0.85 | 1.93 | Undefined | Non-mutagenic | Undefined |
| Isorhamnetin | Good | 316.26 | Optimal | Soluble | No | No | High | 0.55 | 3.26 | Undefined | Undefined | Non-inhibitor |
| Silibinin | Good | 482.44 | Optimal | Soluble | No | No | Low | 0.55 | 4.92 | Undefined | Non-mutagenic | Non-inhibitor |
| Pinoresinol | Good | 358.39 | Optimal | Insoluble | Yes | Yes | High | 0.55 | 3.99 | Undefined | Undefined | Undefined |
| Eugenol | Good | 164.2 | Optimal | Soluble | Yes | No | High | 0.55 | 1.58 | Undefined | Non-mutagenic | Undefined |
| Vanillyl alcohol | Good | 154.16 | Optimal | Soluble | Yes | No | High | 0.55 | 1.26 | Undefined | Non-mutagenic | Non-inhibitor |
| Eupafolin | Good | 316.26 | Optimal | Soluble | No | No | High | 0.55 | 3.18 | Undefined | Undefined | Non-inhibitor |
| Hispidulin | Good | 300.26 | Optimal | Insoluble | No | No | High | 0.55 | 3.12 | Undefined | Undefined | Non-inhibitor |
| Tectorigenin | Good | 300.26 | Optimal | Insoluble | No | No | High | 0.55 | 3.03 | Undefined | Undefined | Non-inhibitor |
| Tetrahydropalmatine | Good | 355.43 | Optimal | Insoluble | Yes | Yes | High | 0.55 | 3.59 | Undefined | Undefined | Undefined |
| Tetrahydropalmatine | Good | 355.43 | Optimal | Insoluble | Yes | Yes | High | 0.55 | 3.59 | Undefined | Undefined | Undefined |
| Jatrorrhizine | Good | 338.38 | Hydrophilic | Insoluble | Yes | Yes | High | 0.55 | 3.06 | Undefined | Undefined | Undefined |
| Palmatine | Good | 352.4 | Optimal | Insoluble | Yes | Yes | High | 0.55 | 3.18 | Unstable in HLM | Undefined | Inhibitor |
| Berberine | Good | 336.36 | Hydrophilic | Insoluble | Yes | Yes | High | 0.55 | 3.14 | Undefined | Undefined | Inhibitor |
| Berberine hydrochloride | Good | 371.81 | Hydrophilic | Insoluble | No | Yes | High | 0.55 | 3.18 | Undefined | Undefined | Inhibitor |
| Oxoglaucine | Good | 351.35 | Optimal | Highly insoluble | Yes | No | High | 0.55 | 3.15 | Undefined | Mutagenic | Undefined |
| Xanthotoxine | Good | 216.19 | Optimal | Soluble | Yes | No | High | 0.55 | 2.97 | Undefined | Undefined | Undefined |
| Bergapten | Good | 216.19 | Optimal | Soluble | Yes | No | High | 0.55 | 2.9 | Undefined | Undefined | Undefined |
| 4-Methoxysalicylaldehyde | Good | 152.15 | Optimal | Soluble | Yes | No | High | 0.55 | 1.14 | Undefined | Undefined | Non-inhibitor |
| Cardamonin | Good | 270.28 | Optimal | Insoluble | Yes | No | High | 0.55 | 2.62 | Undefined | Undefined | Non-inhibitor |
| Leonurine | Good | 311.33 | Optimal | Soluble | No | No | High | 0.55 | 2.75 | Undefined | Non-mutagenic | Undefined |
| Irisflorentin | Good | 386.35 | Optimal | Soluble | No | No | High | 0.55 | 3.78 | Undefined | Undefined | Undefined |
| Deoxypodophyllotoxin | Good | 398.41 | Optimal | Insoluble | Yes | No | High | 0.55 | 4.31 | Undefined | Undefined | Undefined |
| Podophyllotoxin | Good | 414.41 | Optimal | Soluble | No | No | High | 0.55 | 4.64 | Undefined | Non-mutagenic | Undefined |
| Podophyllotoxin glucoside | Bad | 576.55 | Optimal | Soluble | No | Yes | Low | 0.17 | 6.14 | Undefined | Undefined | Undefined |
| Syringin | Good | 372.37 | Hydrophilic | Soluble | No | No | Low | 0.55 | 4.74 | Undefined | Undefined | Non-inhibitor |
| Alpinetin | Good | 270.28 | Optimal | Insoluble | Yes | Yes | High | 0.55 | 3.08 | Undefined | Undefined | Non-inhibitor |
| Acacetin | Good | 284.26 | Optimal | Insoluble | No | No | High | 0.55 | 2.98 | Unstable in HLM | Undefined | Non-inhibitor |
| Formononetin | Good | 268.26 | Optimal | Insoluble | Yes | No | High | 0.55 | 2.81 | Unstable in HLM | Non-mutagenic | Non-inhibitor |
| Biochanin A | Good | 284.26 | Optimal | Insoluble | No | No | High | 0.55 | 2.89 | Unstable in HLM | Undefined | Non-inhibitor |
| Agrimonolide | Good | 314.33 | Optimal | Insoluble | Yes | No | High | 0.55 | 3.3 | Undefined | Undefined | Non-inhibitor |
| Tylophorinidine | Good | 365.42 | Optimal | Soluble | Yes | Yes | High | 0.55 | 3.5 | Undefined | Undefined | Undefined |
| Homopterocarpin | Good | 284.31 | Optimal | Highly insoluble | Yes | Yes | High | 0.55 | 3.64 | Undefined | Undefined | Undefined |
| Limettin | Good | 206.19 | Optimal | Soluble | Yes | No | High | 0.55 | 2.78 | Undefined | Mutagenic | Non-inhibitor |
| Quinidine | Good | 324.42 | Optimal | Soluble | Yes | No | High | 0.55 | 4.34 | Undefined | Non-mutagenic | Undefined |
| Quinine | Good | 324.42 | Optimal | Soluble | Yes | No | High | 0.55 | 4.34 | Undefined | Non-mutagenic | Undefined |
| Cephalotaxine | Good | 315.36 | Optimal | Soluble | Yes | Yes | High | 0.55 | 4.81 | Undefined | Undefined | Undefined |
| Sulforaphane | Good | 177.29 | Optimal | Soluble | No | No | High | 0.55 | 3.07 | Undefined | Mutagenic | Undefined |
