# Supplementary material for: Computation-Based Discovery of Potential Targets for Rheumatoid Arthritis and Related Molecular Screening and Mechanism Analysis of Traditional Chinese Medicine
Source: Dis Markers. 2022 Jun 4;2022:1905077. doi: 10.1155/2022/1905077 (PMC9190478; doi:10.1155/2022/1905077)
Supplement: Supplementary 3 — Supporting material 3: the SAB values of these 42 small molecules of TCM. [file 1905077.f3.doc]

| **Supporting material 3**: The S_AB_ values of these 42 small molecules of TCM | | |
| --- | --- | --- |
| TCMSP ID | Name | S_AB_ |
| MOL000006 | Luteolin | -1.9804 |
| MOL000008 | Apigenin | -2.0047 |
| MOL000098 | Quercetin | -2.2115 |
| MOL000173 | Wogonin | -1.9578 |
| MOL000254 | Eugenol | -2.1584 |
| MOL000305 | Lauric acid | -2.0758 |
| MOL000358 | beta-Sitosterol | -2.1238 |
| MOL000390 | Daidzein | -1.9441 |
| MOL000392 | Formononetin | -1.9559 |
| MOL000422 | Kaempferol | -2.1321 |
| MOL000475 | Anethole | -1.8617 |
| MOL000481 | Genistein | -2.0293 |
| MOL000511 | Ursolic acid | -1.9336 |
| MOL000635 | Vanillin | -1.5166 |
| MOL000666 | Hexanal | -1.2159 |
| MOL002008 | Myricetin | -1.9261 |
| MOL002268 | Rhein | -1.3667 |
| MOL002467 | Gingerol | -1.9005 |
| MOL002773 | beta-Carotene | -1.8481 |
| MOL003186 | Tripterine | -1.6863 |
| MOL003187 | Triptolide | -1.9822 |
| MOL003403 | Nicotine | -2.2397 |
| MOL003493 | Naphthalene | -1.3877 |
| MOL003973 | Caffeine | -2.0319 |
| MOL004932 | Uralsaponin A | -1.7050 |
| MOL005828 | Nobiletin | -1.8117 |
| MOL005916 | Irisolidone | -1.9078 |
| MOL006214 | Progesterone | -2.1424 |
| MOL006356 | Sorbitol | -1.8628 |
| MOL006505 | (-)-Epicatechin | -2.0425 |
| MOL006821 | (-)-Epigallocatechin gallate | -2.0591 |
| MOL007154 | Tanshinone IIA | -2.0517 |
| MOL007990 | Militarin | -1.3246 |
| MOL008680 | Acetaldehyde | -2.2512 |
| MOL008842 | Chenodeoxycholic acid | -1.6364 |
| MOL009357 | Yakuchinone A | -1.3159 |
| MOL009358 | Yakuchinone B | -1.5787 |
| MOL011865 | Rosmarinic acid | -1.9735 |
| MOL012297 | Puerarin | -2.0250 |
| MOL012744 | Resveratrol | -2.1373 |
| MOL013179 | Fisetin | -1.8619 |
| MOL002441 | Dioscin | -1.1638 |
